# The toughest animals of the Earth versus global warming: Effects of long‐term experimental warming on tardigrade community structure of a temperate deciduous forest

**DOI:** 10.1002/ece3.7816

**Published:** 2021-06-29

**Authors:** Matteo Vecchi, Laurent Kossi Adakpo, Robert R. Dunn, Lauren M. Nichols, Clint A. Penick, Nathan J. Sanders, Lorena Rebecchi, Roberto Guidetti

**Affiliations:** ^1^ Department of Biological and Environmental Science University of Jyvaskyla Jyvaskyla Finland; ^2^ Department of Life Sciences University of Modena and Reggio Emilia Modena Italy; ^3^ Department of Applied Ecology North Carolina State University Raleigh NC USA; ^4^ Center for Evolutionary Hologenomics University of Copenhagen Copenhagen Denmark; ^5^ Department of Ecology, Evolution, and Organismal Biology Kennesaw State University Kennesaw GA USA; ^6^ Department of Ecology and Evolutionary Biology University of Michigan Ann Arbor MI USA

**Keywords:** climate change, experimental, global warming, Tardigrades, water bears

## Abstract

Understanding how different taxa respond to global warming is essential for predicting future changes and elaborating strategies to buffer them. Tardigrades are well known for their ability to survive environmental stressors, such as drying and freezing, by undergoing cryptobiosis and rapidly recovering their metabolic function after stressors cease. Determining the extent to which animals that undergo cryptobiosis are affected by environmental warming will help to understand the real magnitude climate change will have on these organisms. Here, we report on the responses of tardigrades within a five‐year‐long, field‐based artificial warming experiment, which consisted of 12 open‐top chambers heated to simulate the projected effects of global warming (ranging from 0 to 5.5°C above ambient temperature) in a temperate deciduous forest of North Carolina (USA). To elucidate the effects of warming on the tardigrade community inhabiting the soil litter, three community diversity indices (abundance, species richness, and Shannon diversity) and the abundance of the three most abundant species (*Diphascon pingue*, *Adropion scoticum*, and *Mesobiotus* sp.) were determined. Their relationships with air temperature, soil moisture, and the interaction between air temperature and soil moisture were tested using Bayesian generalized linear mixed models. Despite observed negative effects of warming on other ground invertebrates in previous studies at this site, long‐term warming did not affect the abundance, richness, or diversity of tardigrades in this experiment. These results are in line with previous experimental studies, indicating that tardigrades may not be directly affected by ongoing global warming, possibly due to their thermotolerance and cryptobiotic abilities to avoid negative effects of stressful temperatures, and the buffering effect on temperature of the soil litter substrate.

## Graphical abstract

Tardigrades are well known for their ability to survive harsh environmental conditions; however, it is not known if they have the potential to survive global warming. We tested the ability of leaf litter tardigrades to resist future warming through a long‐term experimental warming approach. We failed to find any significant effect of warming on leaf litter tardigrades community, suggesting their potential to resist global warming.

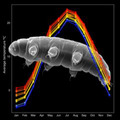

## 1 INTRODUCTION

**FIGURE 1:**
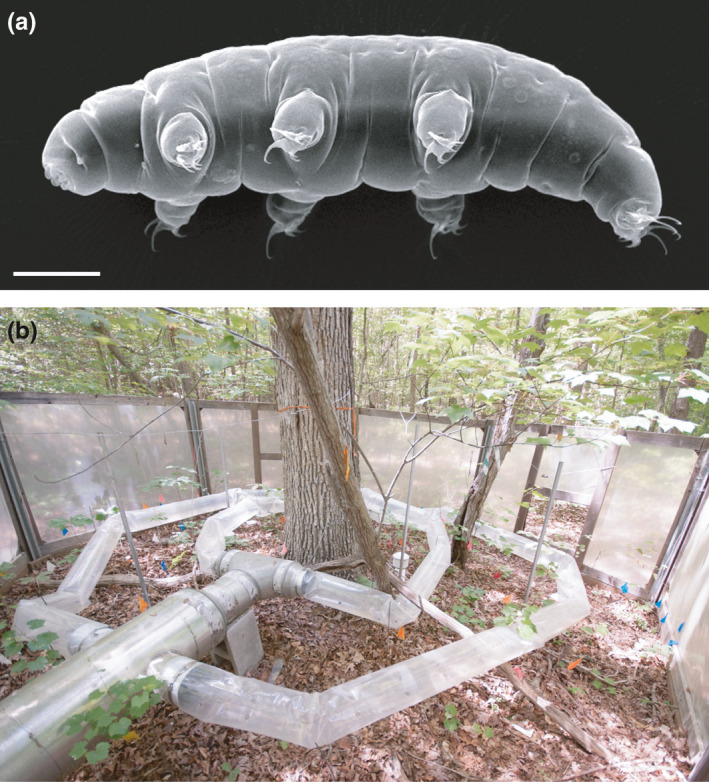
(a) Tardigrade of the genus *Milnesium* (SEM; scale bar = 50 µm). (b) One open‐top chamber experimental setup

Soil organisms such as nematodes, tardigrades, and rotifers—a microfauna that needs a film of water surrounding the body to be active—are generally abundant in most terrestrial biomes. They are relatively poorly studied, especially regarding their response to ongoing climatic changes. Most of the studies concerning the effect of experimental warming on this type of fauna are mainly focused on Antarctic nematode communities (e.g. Andriuzzi et al., 2018; Convey & Wynn‐Williams, 2002; Newsham et al., 2020; Prather et al., 2019; Simmons et al., 2009), and contrasting results on their community have been found according to species and temperature increase (see Section 4). On one hand, these organisms might be particularly susceptible to changes in climate because their surface area relative to volume is great. On the other hand, some of these organisms have life stages that could buffer them from climatic changes. For example, tardigrades (water bears; Figure 1a) have the ability to enter ametabolic physiological states in all phases of their life cycle, allowing them to survive harsh environmental stressors such as desiccation (anhydrobiosis) and freezing (cryobiosis) (Guidetti et al., 2011). The ability of tardigrades to tolerate chemical and physical extremes in an anhydrobiotic state is so extraordinary (Rebecchi et al., 2007) that tardigrades have been called the “toughest animals on the Earth” (Copley, 1999). Though their densities in soil and leaf litter can vary by several orders of magnitude (from 300 up to 33,600 animals/m^2^; Guidetti et al., 1999; Hohberg, 2006), they are commonly detected in leaf litter (Guil & Sanchez‐Moreno, 2013) on all continents (Guidetti et al., 1999).

Previous studies on the impacts of climate changes on tardigrade communities have found somewhat surprising results: no effect of increasing temperature on tardigrade communities has been detected (Briones et al., 1997; Sohlenius & Boström, 1999a). To better understand the effects of increasing air temperature on tardigrade communities of soil litter, we sampled a tardigrade community within an extensive set of experimental warming arrays in the eastern United States, performed in the Duke Forest in the Piedmont region of North Carolina (Pelini et al., 2011). Experimental warming has the potential to analyze the response of the entire tardigrade community compared to transplant experiments because the warming is performed in situ without the need to move the animals. Moreover, the use of open‐top chambers minimizes the substrate disturbance and allows for long‐term, consistent warming over a sustained period (Pelini et al., 2011). Previous studies in the experimental warming array used in this study (Figure 1b) found that warming had differential effects on ants, other ground‐living arthropods, and microbial communities, with some species benefiting from warming and others declining. In particular, ants have species‐specific responses to temperature increase with some species benefiting from warming and others declining probably linked to physiological traits that differ between species (Diamond et al., 2013, 2016; MacLean et al., 2017; Pelini et al., 2014; Penick et al., 2017), and the same is true for other ground‐living arthropods (Fitzgerald et al., 2021), while the effect of warming on microbial community structure and function may become more pronounced as soil temperatures increase and carbon substrates are depleted through time at the Duke Forest site but were not affected at a more northern site where this experiment was replicated (Cregger et al., 2014).

The experimental design allowed the opportunity to test for the interactive effects between air warming and other environmental variables, such as soil moisture. We hypothesized two possible outcomes of this experiment: (a) we predicted that the tardigrade community (or part of it) would be influenced by both increasing temperature and soil moisture with regard to abundance (positive correlation between increased temperature and moisture on tardigrade abundances) and community composition (change in dominant species and/or diversity); (b) alternatively, we predicted that the tardigrade community (or part of it) will show no change in responses to the warming treatment, because they escape negative impacts of warming due to anhydrobiosis (avoiding period of substrate drying) or due to an extreme tolerance of high temperatures (see Giovannini et al., 2018; Li & Wang, 2005; Neves et al., 2020; Rebecchi, Boschini, et al., 2009). Those predictions come from the general trend observed for the effect of artificial warming on other terrestrial organisms belonging to the hydrobios, as the soil nematodes, that share the same habitat with tardigrades (Bakonyi & Nagy, 2000; Hiltpold et al., 2017; Yan et al., 2017).

## 2 MATERIALS AND METHODS

### 2.1 Experimental design and warming chambers

**FIGURE 2:**
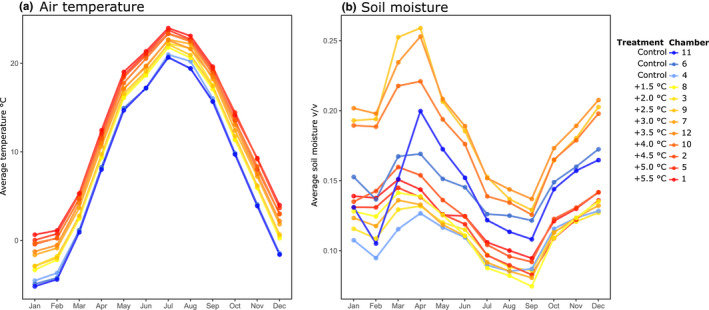
(a) Average air temperature by month over 5 years of the experimental chambers. (b) Average soil moisture by month over 5 years of the experimental chambers

The experimental facilities used in this study are described in Pelini et al. (2011). These facilities were established in a relatively warm site at Duke Forest near Hillsborough (35°52′00″N, 79°59′45″W, 130 m a.s.l.) in the Piedmont region of North Carolina (USA). The site is located near the center of the deciduous forest biome in eastern North America. The experimental site at Duke Forest was in a ca. 80‐year‐old oak‐hickory stand within the Eno River Unit. The mean annual temperature at Duke Forest is 15.5°C, and the mean annual precipitation is 1,140 mm. The site had 12 octagonal open‐top chambers (each one 5 m in diameter with eight walls each 1.9 m wide and 1.2 m tall with an area of 17.4 m^2^), coded with numbers from 1 to 12, that warmed the forest floor year‐round with thermostat‐controlled forced air passed over hydronic heaters (Figure 1b). Nine chambers were warmed with a different target temperature each (from 1.5 to 5.5°C above ambient temperature with increments of 0.5°C). The heating of the chambers was dynamically adjusted based on the external environment temperature to obtain a constant increment over the environment temperature (Figure 2a). The highest temperature increase of 5.5°C was included to mimic the worst scenario predicted for the year 2100 (RCP 8.5; IPCC, 2013). Three chambers considered as controls had air blown at environment temperature (no heat). The experiment lasted for five years (2010–2015).

### 2.2 Leaf litter sampling and tardigrade extraction

Leaf litter sampling was conducted on May 15, 2015. Two samples (each one of a volume of about 0.5 dm^3^) of the superficial layer of leaf litter (~10 cm of depth, composed by a variety of leaves of tree plant species but dominated by *Quercus* *alba* L.) were collected from two random points from each of the 12 chambers (nine chambers with increasing air temperatures and three control chambers), for a total of 24 samples. Samples were air‐dried immediately after collection and shipped in zip‐locked plastic bags to the Department of Life Sciences of University of Modena and Reggio Emilia (UNIMORE) in Modena (Italy). The leaf litter was kept desiccated for 2 years until tardigrade extraction. Before animal extraction, desiccated leaf litter was kept at 45% relative humidity (RH) at 20°C for 24 hr, and then, 10 g of each sample (pseudoreplicate) for each chamber were rehydrated in tap water for 30 min. We then sieved the leaf litter and water using two sieves of different mesh sizes: 500 μm and 37 μm. The leaf litter left in the 500 μm sieve was resubmerged in water and sieved a second time after 2 hr to be sure to extract all tardigrades and their eggs. The debris collected from the 37 μm sieve was resuspended in distilled water and tardigrades along with their eggs were collected manually with a glass pipette under a stereomicroscope.

### 2.3 Species identification and data analysis

The tardigrades were fixed in Carnoy's fixative (¾ methanol, ¼ acetic acid) and mounted on microscopic slides in Faure's mounting medium. Animal observations were carried out with light microscopy (LM) under phase contrast (PhC) and differential interference contrast (DIC) up to the maximum magnification (100× oil objective) with a Leica DM RB microscope, at the Laboratory of Evolutionary Zoology (UNIMORE). Eggs were not included in the analyses due to their low number. Tardigrade classification was mainly done by following Ramazzotti and Maucci (1983) and Bingemer and Hohberg (2017).

Tardigrades are very sensitive to the moisture of the substrates being aquatic animals (although able to live in terrestrial substrates); soil moisture is one of the main environmental factors that could be effected by the air temperature increase. For these reasons, in addition to chamber air temperature, soil moisture was chosen as an additional variable. In this specific experimental setup, soil temperature is correlated with air temperature (Cregger et al., 2014) but soil moisture is not correlated with the air temperature measures (Burt et al., 2014; Figure 2b). We chose to present the analysis done with air temperature as independent variable instead of soil temperature because within the experimental setup, air temperature was the directly manipulated variable and soil temperature was then causally dependent on it. We also performed the analysis with organic soil temperature instead of air temperature, and the results were very similar (analysis with soil temperature is available as Appendices S1 and S2). Average air temperature and soil moisture were calculated from the raw data file in Ellison and Dunn (2017); for statistical analysis, the 5‐years average of each chamber was used.

Three community indices considering all tardigrade species (abundance, species richness, and Shannon index) and the abundance of the three most abundant tardigrade species [*Diphascon pingue* (Marcus, 1936), *Adropion scoticum* (Murray, 1905), and *Mesobiotus* sp.] were examined and related to the artificially increased air temperatures and soil moisture (singly and in pair). Diversity index calculations and variance partitioning were performed with R software (base package, stats package, vegan package; Oksanen et al., 2018). Variance partitioning was performed to compare the variance contribution of the pseudoreplicates within samples by means of the “aov” function from the R base package with a nested design, without considering the significance testing. Differences in specimen abundances (of all species and the three individual species), number of species (Species richness), and Shannon index were tested against air temperature, soil moisture, and their interaction using Bayesian generalized linear mixed models (GLMMs) implemented in JAGS with the R package R2jags (Su & Yajima, 2012). In all models, chamber identity (i.e., each individual chamber code) was used as a random effect. Predictors (air temperature and soil moisture) were scaled and centered before model fitting. For all three response variables, a log link function was used. The error families used were negative binomial, Poisson, and Gamma for number of specimens, number of species, and Shannon index, respectively. From the posterior distributions of the model parameter estimates, a Bayesian *p*‐value and standardized effect sizes were calculated according to Makowski et al. (2019) and Cohen (1988). All of the analyses performed with their code are reported in Appendix S1.

## 3 RESULTS

Each of the 24 samples of leaf litter contained tardigrades. A total of 1,762 tardigrades, belonging to 13 taxa, were collected, for a mean of 14.6 (min 6, max 40) tardigrades/g of dry leaf litter (Table 1). Three species, namely *Diphascon pingue*, *Adropion scoticum,* and *Mesobiotus* sp., were found to be numerically dominant, accounting for more than 95% of all observed individuals (41.03%, 40.69%, and 13.96%, respectively). One species (*Pseudechiniscus* sp.) was a singleton, found as a single individual in a single chamber (Appendix S3).

**TABLE 1:** Average temperature and soil moisture of each chamber (from 5 years of measuring) and the tardigrades abundance in the leaf litter

Chamber	Average air temperature (°C)	Average soil moisture (v/v)	Average abundance of tardigrades (per 10 gram sample of leaf litter)	Standard deviation of tardigrade abundance (per 10 gram sample of leaf litter)
1	12.9443	0.1267	32.5	26.16
2	12.2119	0.1290	122.0	56.57
3	10.2466	0.1133	44.5	9.19
4	8.6845	0.1087	84.5	103.95
5	12.6972	0.1196	43.5	31.82
6	8.4891	0.1485	28.5	23.34
7	11.2096	0.1144	47.0	25.46
8	9.9831	0.1158	112.0	131.52
9	10.3387	0.1894	31.5	4.95
10	12.1041	0.1784	79.5	65.76
11	8.4038	0.1445	53.5	65.76
12	11.4322	0.1919	202.0	206.48

Note

Underlined chambers are controls (i.e., chambers without heating).

The variance partition (Table 2) between pseudoreplicates within each chamber and among chambers showed that most of the variance for all examined indexes was best explained by the differences among pseudoreplicates of the same chamber than the difference among different chambers (e.g., for *D*. *pingue* individuals, this difference explains almost 100% of the variation). These high values of variance shown by pseudoreplicates indicate a very high small‐scale heterogeneity (at grains less than 5 m) in the distribution of tardigrades. The Bayesian GLMMs converged with good Rhat and autocorrelation values (see Appendixs S1).

**TABLE 2:** Variance partitioning and Bayesian GLMM results

	Abundance	Taxa	Shannon	*Adropion*	*Mesobiotus*	*Diphascon*
Variance partitioning						
Variance between pseudoreplicates/total variance	94.85%	94.08%	99.20%	91.49%	99.14%	99.91%
Bayesian GLMM results						
Air temperature	0.369/0.003	0.927/0.007	0.788/0.073	0.715/0.004	0.515/0.015	0.522/0.004
Soil moisture	0.886/0.000	0.422/0.059	0.979/−0.007	0.510/0.008	0.141/−0.039	0.753/−0.002
Temperature*Moisture	0.086/0.008	0.778/−0.029	0.255/**−0.472**	0.728/−0.007	0.088/0.063	**0.049**/0.021

Note

Existence (Bayesian *p*‐value (*p*)) and effect size statistics (directional median Cohen's *d* (*d*)) of the effect of the environmental parameters (air temperature, soil moisture, air temperature * soil moisture) on the community structure parameters (abundance = total number of tardigrades, taxa = total number of species, Shannon = Shannon index, *Adropion* = number of animals of *Adropion scoticum*, *Diphascon* = number of animal of *Diphascon pingue*, *Mesobiotus* = number of animal of *Mesobiotus* sp. Statistics are presented as *p*/*d*. In bold: *p* < 0.05, |*d*| > 0.1.

Bayesian *p*‐values and effect sizes (Table 2) suggest the absence of an effect for all the combinations of predictors and response variables. The effect of the interaction of temperature and moisture on *Diphascon* has a *p*‐value slightly below 0.05; however, the effect size of 0.049 suggests that even if the effect exists, it would be so small to not be of biological relevance. Inversely, the effect of the interaction of air temperature and moisture on the Shannon index shows a high absolute value of effect size (−0.472); however, it is not possible to confirm whether this effect exists as the Bayesian *p*‐value is 0.255. Additionally, in all posterior estimates of the regression parameters, the 95% highest density interval (HDI) comprises the value 0 (Appendix S1).

This can be translated in the absence of a significant effect of the predictors tested (i.e., air temperature, soil moisture, and their interaction). In other words, none of the three diversity indexes (number of specimens, species richness, and Shannon index) nor the abundances of the three species tested individually were correlated with mean chamber measurements of air temperature values, soil moisture, or air temperature and soil moisture when considered together (i.e., the synergic/cross related effects of these environmental parameters) after the 5 years of the experiment. To summarize, no significant influence of the long‐term warming environmental treatments was found on the examined ecological indexes of the tardigrade community.

## 4 DISCUSSION

We found that 5 years of experimental warming had no detectable effect on the tardigrade community (nor on a specific component of it) inhabiting the soil litter. While warming has generally affected community composition of those taxa (i.e., bacteria, ants, and other ground arthropods) studied within the context of the Duke Forest warming experiment (Cregger et al., 2014; Diamond et al., 2016; Fitzgerald et al., 2021; Pelini et al., 2014), our results for tardigrades are unique in that we observed no effects of the warming treatment. This was true even in the warmest chambers in which temperatures were raised to mimic the worst scenario predicted (RCP 8.5; IPCC, 2013) for the year 2100.

Our results are in line with other studies that have failed to find an effect of warming or other manipulations on tardigrade communities. Sohlenius and Boström (1999a), for example, found no differences in tardigrade abundance between the treatments (samples transplanted to other climatic conditions). Their work was based on short‐term transplant studies lasting around one year during which temperature and rainfall changed but also the surrounding substrate. On the other hand, in another short‐term transplant study, Briones et al. (1997) found that tardigrade population oscillations were positively correlated with temperature, though only in the colder sites.

There are difficulties in comparing the results of the studies related to the effects of environmental warming on terrestrial microfauna (e.g., tardigrades vs nematodes) due to the differences in the experimental designs used in those studies. For example, the studies on nematodes, that are those performed more extensively, have been conducted: in different geographic areas, ecotypes, and habitats, e.g., bryophytes (Newsham et al., 2020), acid soil (Stevnbak et al., 2012), temperate‐semiarid soil (Bakonyi & Nagy, 2000; Yan et al., 2017), temperate‐boreal forest soil (Thakur et al., 2014), and tundra (Sohlenius & Bostrom, 1999b); moreover, most of them were performed in Antarctic soils (Andriuzzi et al., 2018; Convey & Wynn‐Williams, 2002; Knox et al., 2017; Simmons et al., 2009) where the effects of warming are difficult to separate from those of the freeze–thaw cycles (Knox et al., 2017; Simmons et al., 2009); using different experimental approaches, e.g., transplants (Sohlenius & Boström, 1999a, 1999b), greenhouses (Convey & Wynn‐Williams, 2002; Stevnbak et al., 2012), open‐top chambers (Newsham et al., 2020; Prather et al., 2019), or different heating systems (e.g. infrared [Yan et al., 2017], ceramic and cable heaters [Thakur et al., 2014], and sun reflection [Bakonyi & Nagy, 2000]); to last for different times, e.g., months (Bakonyi & Nagy, 2000) or years (Newsham et al., 2020); and targeting different taxa (e.g. Andriuzzi et al., 2018; Convey & Wynn‐Williams, 2002). These studies performed in different conditions showed some contrasting results of the effects of warming on nematode communities: for example, reduction (Simmons et al., 2009; Stevnbak et al., 2012; Yan et al., 2017) and/or increase (Blankinship et al., 2011; Sohlenius & Bostrom, 1999b) in abundances, or different responses according to the species and/or trophic level (see Hiltpold et al., 2017). Therefore, soil nematode communities show responses to climate change that are context‐dependent and are moderated by ecosystem characteristics such as vegetation type and/or nutrient levels (Hiltpold et al., 2017; Prather et al., 2019). Moreover, for most of the microfauna species considered in this and previous studies (e.g., tardigrades and nematodes), very little is known about their ecology and microhabitats conditions.

Our findings come from a single tardigrade community from a single location; they may indicate that tardigrades are resilient to the increases in temperature. The comparisons of our results with those obtained with nematodes, with which share similar size, anhydrobiotic capabilities, and often existing in similar substrates, can be difficult due to the abovementioned reasons. Anyway, in an experiment conducted in a similar habitat (i.e., temperate forest), the effects of 2 years increased soil temperature recorded on nematode community were similar to those found in our experiment: no effect on total or trophic group abundances (Thakur et al., 2014).

We have three hypotheses, that are not necessarily mutually exclusive, that could explain the lack of response of tardigrades to extreme experimental warming. First, the tardigrades at the study site may have unusually high thermal optima and maxima, such that they thrive even as conditions warm. Even tardigrade species living in cold regions appear to have relatively high thermal limits. For example, the Antarctic species *Acutuncus antarcticus* (Richters, 1904) can withstand short heat shocks up to 33°C (Giovannini et al., 2018). Similarly, the boreo‐alpine tardigrade species *Borealibius zetlandicus* (Murray, 1907) showed a lethal temperature (LT50) of 33°C (Rebecchi, Boschini, et al., 2009). It seems plausible that the thermal limits of tardigrades living in the far warmer conditions of the southern part of North America would be higher and perhaps high enough to allow success even in light of the warming studied here.

Second, the resistance of tardigrades to warming may be further explained by the characteristics of their habitat. The top layer of soil, the upper horizon, is made up of living and decomposed materials (e.g., leaf litter); the presence of wide air‐filled spaces and its heterogeneity can buffer the increase of external temperature and allow animals to select the most suitable microhabitat. Tardigrade responses to small‐scale habitat heterogeneity (patchiness) could explain the high variability among pseudoreplicates as shown to be the predominant source of variance for all six examined indexes (Table 2). Leetham et al. (1982) and Hohberg (2006) also found a high intrasite variance in the number of tardigrades in soil habitats, and a similar level of patchiness has been shown for other substrates as well (Meyer, 2006; Sohlenius et al., 2004; Tilbert et al., 2019). In our experimental setup, soil moisture was not correlated with air temperature and was instead correlated with air relative humidity (Burt et al., 2014). The difference between soil moisture and air humidity is likely to create a moisture gradient in the leaf litter, generating microhabitats with different moisture levels. The potential indirect effect of warming (moisture change) could then be buffered by tardigrades moving to or living in different leaf litter areas/layers with a more suitable moisture, as tardigrades have been shown to be able to differentially colonize different soil and leaf litter layers according to the seasons (Briones et al., 1997; Guidetti et al., 1999, respectively).

Third, the ability of tardigrades to enter anhydrobiosis (Guidetti et al., 2011). The thermal tolerance of tardigrades in their desiccated state is known to be higher than when they are in their active state (Neves et al., 2020; Rebecchi, Cesari, et al., 2009). It is possible that tardigrades escaped the negative effects of high temperatures in our study, especially during summer; because they were in a desiccated state of anhydrobiosis, they could compensate the larger amount of time spent in anhydrobiosis in summer by taking advantage of a reduced frozen period in winter. During summer when temperatures peak in temperate environments, water availability is usually reduced in substrates (leaf litter, mosses, and lichens) inhabited by tardigrades, which dry out quickly. It is thus possible that, in our study site, tardigrades are most active in cooler times of year and spend the hottest time of the year in a state of anhydrobiosis, a state in which their thermal tolerance is increased (Neves et al., 2020). The co‐occurrence of warmer temperatures with dry conditions could be an additional factor that increases tardigrade survival to warming in specific climatic areas.

The experimental data obtained in this work represent a small light to illuminate the effects of climate changes in the dark world of soil microfauna. It highlights that tardigrades in soil litter are part of those tolerant species able to survive the increase in air temperature due to global warming, and this may be related to their ability to enter anhydrobiosis or a higher tolerance of thermal extremes.

## CONFLICT OF INTERESTS

The authors have no competing interests to declare.

## AUTHOR CONTRIBUTIONS

**Matteo Vecchi:** Conceptualization (equal); Data curation (equal); Formal analysis (lead); Investigation (equal); Methodology (equal); Visualization (lead); Writing‐original draft (lead); Writing‐review & editing (equal). **Laurent** **Kossi Adakpo:** Data curation (equal); Investigation (equal). **Robert R. Dunn:** Conceptualization (equal); Funding acquisition (equal); Project administration (lead); Resources (equal); Supervision (equal); Writing‐review & editing (equal). **Lauren M. Nichols:** Conceptualization (equal); Data curation (equal); Funding acquisition (equal); Resources (equal); Writing‐review & editing (equal). **Clint A**. **Penick:** Conceptualization (equal); Data curation (equal); Funding acquisition (equal); Resources (equal); Writing‐review & editing (equal). **Nathan J. Sanders:** Conceptualization (equal); Data curation (equal); Funding acquisition (equal); Resources (equal); Writing‐review & editing (equal). **Lorena** **Rebecchi:** Funding acquisition (equal); Supervision (equal); Writing‐review & editing (equal). **Roberto** **Guidetti:** Conceptualization (equal); Funding acquisition (equal); Resources (equal); Supervision (equal); Writing‐original draft (supporting); Writing‐review & editing (equal).

## Supporting information

Appendix S1‐S2

Appendix S3

## Data Availability

Warming chamber environmental variables are available at: http://harvardforest.fas.harvard.edu:8080/exist/xquery/data.xq?id=hf113. Appendices [Link], [Link] and a copy of the raw environmental measurements are also available in Figshare under the https://doi.org/10.6084/m9.figshare.14742699.
