# Supplementary material for: The toughest animals of the Earth versus global warming: Effects of long‐term experimental warming on tardigrade community structure of a temperate deciduous forest
Source: Ecol Evol. 2021 Jun 29;11(14):9856–63. doi: 10.1002/ece3.7816 (PMC8293726; doi:10.1002/ece3.7816)
Supplement: Supplementary file 1 — Appendix S1‐S2 [file ECE3-11-9856-s002.zip › SM.01.html]

Tardigrades in warming chambers - Air temperature


# Tardigrades in warming chambers - Air temperature

#### 2020-10-01

Data analysis of the “Tardigrades in Duke forest warming chambers project” for the publication: Vecchi M., Kossi Adakpo L., Dunn R.R., Nichols L.M., Penick C.A., Sanders N.J., Rebecchi L., Guidetti R. (2021). The toughest animals of the Earth versus global warming: Effects of long‐term experimental warming on tardigrade community structure of a temperate deciduous forest. *Ecology And Evolution*. Supplementary file S1.

Load all the libraries I need and get the info on the session with all the packages versions

```
library(R2jags) #running the models
library(tidyverse)
library(tidyr)  #sorting the models outputs for plotting
library(ggplot2) #plotting
library(ggridges) #plotting
library(DT) #show interactive table in the html file
library(vegan)
library(bayestestR)
library(robustbase)
library(patchwork)
sessionInfo()
```

```
## R version 4.0.2 (2020-06-22)
## Platform: x86_64-apple-darwin17.0 (64-bit)
## Running under: macOS  10.16
## 
## Matrix products: default
## BLAS:   /Library/Frameworks/R.framework/Versions/4.0/Resources/lib/libRblas.dylib
## LAPACK: /Library/Frameworks/R.framework/Versions/4.0/Resources/lib/libRlapack.dylib
## 
## locale:
## [1] en_US.UTF-8/en_US.UTF-8/en_US.UTF-8/C/en_US.UTF-8/en_US.UTF-8
## 
## attached base packages:
## [1] stats     graphics  grDevices utils     datasets  methods   base     
## 
## other attached packages:
##  [1] patchwork_1.1.0   robustbase_0.93-7 bayestestR_0.8.2  vegan_2.5-6      
##  [5] lattice_0.20-41   permute_0.9-5     DT_0.17           ggridges_0.5.2   
##  [9] forcats_0.5.0     stringr_1.4.0     dplyr_1.0.0       purrr_0.3.4      
## [13] readr_1.3.1       tidyr_1.1.0       tibble_3.0.1      ggplot2_3.3.2    
## [17] tidyverse_1.3.0   R2jags_0.6-1      rjags_4-10        coda_0.19-3      
## 
## loaded via a namespace (and not attached):
##  [1] httr_1.4.2        jsonlite_1.7.2    splines_4.0.2     modelr_0.1.8     
##  [5] assertthat_0.2.1  blob_1.2.1        cellranger_1.1.0  yaml_2.2.1       
##  [9] R2WinBUGS_2.1-21  pillar_1.4.4      backports_1.1.8   glue_1.4.1       
## [13] digest_0.6.25     rvest_0.3.5       colorspace_1.4-1  htmltools_0.5.1.1
## [17] Matrix_1.2-18     plyr_1.8.6        pkgconfig_2.0.3   broom_0.5.6      
## [21] haven_2.3.1       scales_1.1.1      mgcv_1.8-31       generics_0.1.0   
## [25] ellipsis_0.3.1    withr_2.4.1       cli_2.3.1         magrittr_1.5     
## [29] crayon_1.3.4      readxl_1.3.1      evaluate_0.14     fs_1.4.2         
## [33] nlme_3.1-148      MASS_7.3-51.6     xml2_1.3.2        tools_4.0.2      
## [37] hms_0.5.3         lifecycle_0.2.0   munsell_0.5.0     reprex_0.3.0     
## [41] cluster_2.1.0     compiler_4.0.2    rlang_0.4.10      grid_4.0.2       
## [45] rstudioapi_0.11   htmlwidgets_1.5.1 rmarkdown_2.3     boot_1.3-25      
## [49] gtable_0.3.0      abind_1.4-5       DBI_1.1.0         R6_2.4.1         
## [53] lubridate_1.7.9   knitr_1.29        insight_0.12.0    stringi_1.4.6    
## [57] parallel_4.0.2    Rcpp_1.0.4.6      vctrs_0.3.1       DEoptimR_1.0-8   
## [61] dbplyr_1.4.4      tidyselect_1.1.0  xfun_0.15
```

##Environmental measurements To get the chambers mean temperature and soil moisture download the raw measurements file hf113-02 from: http://harvardforest.fas.harvard.edu:8080/exist/apps/datasets/showData.html?id=hf113

###Load the environmental measures data

```
environmental_measures=read.csv("hf113-02-hf-chamber-since-2009.csv",header=T)
```

###Check the structure of the data

```
ncol(environmental_measures)
```

```
## [1] 40
```

```
nrow(environmental_measures)
```

```
## [1] 678786
```

```
colnames(environmental_measures)
```

```
##  [1] "datetime"  "year"      "decdate"   "doy"       "month"     "week"     
##  [7] "dom"       "hour"      "date"      "chamber"   "cat1.avg"  "cat2.avg" 
## [13] "catd.avg"  "csto1.avg" "csti1.avg" "csto2.avg" "csti2.avg" "cq.avg"   
## [19] "crh.avg"   "csm.avg"   "cat1.min"  "cat2.min"  "catd.min"  "csto1.min"
## [25] "csti1.min" "csto2.min" "csti2.min" "cq.min"    "crh.min"   "csm.min"  
## [31] "cat1.max"  "cat2.max"  "catd.max"  "csto1.max" "csti1.max" "csto2.max"
## [37] "csti2.max" "cq.max"    "crh.max"   "csm.max"
```

To be sure that there is nothing strange going on I plot the three column I´m interested in (cat1.avg, cat2.avg, csm.avg).

```
par(mfrow = c(1, 3))
boxplot(environmental_measures$cat1.avg, main = "Boxplot of cat1.avg", ylab = "Temperature Celsius")
boxplot(environmental_measures$cat2.avg, main = "Boxplot of cat2.avg", ylab = "Temperature Celsius")
boxplot(environmental_measures$csm.avg, main = "Boxplot of csm.avg", ylab = "Soil moisture v/v")
```

The data looks OK, so I can select only the variables of my interest

###Get the average measure for each chamber

As there are two temperature measurements for each chamber.

```
environmental_measures$cat.avg = rowMeans(environmental_measures[,11:12], na.rm=T)
```

Now I keep only the columns I am interested in (Chamber, cat.avg, csm.avg).

```
environmental_measures = environmental_measures[,c(10,20,41)]
colnames(environmental_measures)
```

```
## [1] "chamber" "csm.avg" "cat.avg"
```

Now I can average the measures by chamber

```
cat_mean_by_chamber = aggregate(environmental_measures$cat.avg, by = list(environmental_measures$chamber), FUN=mean, na.rm=T)
csm_mean_by_chamber = aggregate(environmental_measures$csm.avg, by = list(environmental_measures$chamber), FUN=mean, na.rm=T)
environmental_means = data.frame(chamber=1:12, cat.avg=cat_mean_by_chamber$x, csm.avg=csm_mean_by_chamber$x)
environmental_means
```

```
##    chamber   cat.avg   csm.avg
## 1        1 12.944333 0.1267617
## 2        2 12.211956 0.1290056
## 3        3 10.246583 0.1133538
## 4        4  8.684541 0.1086629
## 5        5 12.697168 0.1195839
## 6        6  8.489095 0.1484743
## 7        7 11.209632 0.1144070
## 8        8  9.983129 0.1157840
## 9        9 10.338733 0.1894105
## 10      10 12.104089 0.1783753
## 11      11  8.403804 0.1444671
## 12      12 11.432208 0.1919299
```

##Tardigrades dataset Now load the table with the number of individual recorded for each species in each chamber/replicate.

```
tardigrades_table=read.csv("tardigrades_warming_chambers_individuals.csv",header=T)
```

Let´s check the table:

```
head(tardigrades_table)
```

```
##   Chamber Mesobiotus Paramacrobiotus Macrobiotus Adropion Diphascon Hypsibius
## 1       1          1               0           0        1        11         0
## 2       1         16               0           0        2        24         0
## 3       2          9               0           0       66        82         0
## 4       2         13               0           0       54        15         0
## 5       3          4               0           0        0        33         0
## 6       3         21               0           5       12        12         1
##   Minibiotus Isohypsibius Milnesium Echiniscus Pilatobius Itaquascon
## 1          0            0         0          0          0          1
## 2          0            0         0          0          4          5
## 3          2            2         0          1          0          0
## 4          0            0         0          0          0          0
## 5          0            1         0          0          0          0
## 6          0            0         0          0          0          0
##   Pseudechiniscus
## 1               0
## 2               0
## 3               0
## 4               0
## 5               0
## 6               0
```

###Calculate the diversity index of tardigrade community Number of individuals

```
Individuals = rowSums(tardigrades_table[,2:ncol(tardigrades_table)])
```

Number of taxa

```
Taxa = rowSums(tardigrades_table[,2:ncol(tardigrades_table)]>0)
```

Shannon index

```
Shannon = vegan::diversity(tardigrades_table[,2:ncol(tardigrades_table)], index = "shannon")
```

##Let´s put all the data together I put all the environmental measures and community idexes in one table

First I duplicate each row of the environmental\_means table to accomodate for the two replicates for each chamber in the tardigrades table

```
environmental_means = environmental_means[rep(row.names(environmental_means) , 2) , ]
environmental_means = environmental_means[order(environmental_means$chamber),]
```

Then I attach all the data together

```
tardigrades_alldata=cbind(environmental_means,tardigrades_table,Individuals,Taxa,Shannon)
```

Here´s the complete data table

```
DT::datatable(tardigrades_alldata, rownames = FALSE)
```

## Variance partition

Estimate and plot variance partition

```
tardigrades_alldata$replicate=paste0("r",1:24)
anovas=summary(aov(data=tardigrades_alldata,cbind(Individuals,Taxa,Shannon,Mesobiotus,Adropion,Diphascon)~Chamber/replicate))
variances=lapply(anovas,function(x){x$`Sum Sq`})
variances.m=data.frame(matrix(unlist(variances)))
variances.m$level=rownames(anovas[[1]])
variances.m$var=c("Ind","Ind","Tax","Tax","Sha","Sha","Mesobiotus","Mesobiotus","Adropion","Adropion","Diphascon","Diphascon")
colnames(variances.m)[1]="value"
p=ggplot(variances.m)+geom_bar(aes(x=var,y=value,fill=level),position="fill",stat="identity")
p
```

## Bayesian GLMMs

###Individuals

Fort the individuals count i´ll try two different error families: negative binomial and poisson. The link function is log Negative Binomial and Poisson are chosen because they´re discrete probability distributions that goes from 0 to +Inf, like the indidividuals count index.

Let´s create the jags data for both the models:

```
data_jags=list(Nchamber=12,
                chamber=as.factor(tardigrades_alldata$Chamber),
                individuals=tardigrades_alldata$Individuals,
                temperature=as.numeric(scale(tardigrades_alldata$cat.avg)),
                moisture=as.numeric(scale(tardigrades_alldata$csm.avg)))
```

And run them

```
################################################
###### Individuals with Negative Binomial ######
################################################

mod_jags_nb <- function(){
# Priors
    alpha ~ dunif(-1000,1000)# intercept
    beta_temp ~ dunif(-1000,1000)# beta for temperature
    beta_moist ~ dunif(-1000,1000)# beta for soil moisture
    beta_inter ~ dunif(-1000,1000)# beta for interaction
    sigma_a ~ dunif(0, 100) # standard deviation of random effect (variance between sites)
    tau_a <- 1 / (sigma_a * sigma_a) # convert to precision
    r ~ dunif(0,100) # prior for overdispersion parameter  

# Random intercept for each chamber (random effect)
        for (j in 1:Nchamber){
        a[j] ~ dnorm(0, tau_a) 
    }

# Likelihood:
    for (i in 1:length(individuals)){
        mu[i] <- exp(alpha + a[chamber[i]] + beta_temp * temperature[i] + beta_moist * moisture[i] + beta_inter * temperature[i] * moisture[i]) #predicted values with log link
        p[i] <- r / ( r + mu[i]) #reparametrization of negbin parameters according to: http://doingbayesiandataanalysis.blogspot.com/2012/04/negative-binomial-reparameterization.html
        individuals[i] ~ dnegbin( p[i] , r )
    }
}

mod_params_nb <- c("alpha", "beta_temp", "beta_moist", "beta_inter", "sigma_a","r")


mod.nb.ind=jags(data=data_jags,parameters.to.save=mod_params_nb, model.file=mod_jags_nb,n.iter=10000000)

######################################
###### Individuals with Poisson ######
######################################

mod_jags_pois <- function(){
# Priors
    alpha ~ dunif(-1000,1000)# intercept
    beta_temp ~ dunif(-1000,1000)# beta for temperature
    beta_moist ~ dunif(-1000,1000)# beta for soil moisture
    beta_inter ~ dunif(-1000,1000)# beta for interaction
    sigma_a ~ dunif(0, 100) # standard deviation of random effect (variance between sites)
    tau_a <- 1 / (sigma_a * sigma_a) # convert to precision

# Random intercept for each chamber (random effect)
        for (j in 1:Nchamber){
        a[j] ~ dnorm(0, tau_a) 
    }

# Likelihood:
    for (i in 1:length(individuals)){
        mu[i] <- exp(alpha + a[chamber[i]] + beta_temp * temperature[i] + beta_moist * moisture[i] + beta_inter * temperature[i] * moisture[i]) #predicted values with log link
        individuals[i] ~ dpois(mu[i])
    }
}

mod_params_pois <- c("alpha", "beta_temp", "beta_moist", "beta_inter", "sigma_a")


mod.pois.ind=jags(data=data_jags,parameters.to.save=mod_params_pois, model.file=mod_jags_pois,n.iter=10000000)
```

Now check if the models converged and their DICS

```
mod.nb.ind
```

```
## Inference for Bugs model at "C:/Users/mavecchi/AppData/Local/Temp/RtmpULkW5y/model2dc7fd158cd.txt", fit using jags,
##  3 chains, each with 1e+07 iterations (first 5e+06 discarded), n.thin = 5000
##  n.sims = 3000 iterations saved
##            mu.vect sd.vect    2.5%     25%     50%     75%   97.5%  Rhat n.eff
## alpha        4.221   0.222   3.798   4.070   4.218   4.359   4.685 1.001  3000
## beta_inter   0.634   0.382  -0.083   0.390   0.625   0.875   1.404 1.001  3000
## beta_moist   0.033   0.243  -0.455  -0.117   0.029   0.184   0.526 1.001  3000
## beta_temp    0.199   0.245  -0.285   0.053   0.204   0.350   0.674 1.002  1600
## r            1.468   0.438   0.771   1.158   1.407   1.723   2.447 1.001  3000
## sigma_a      0.356   0.282   0.015   0.149   0.301   0.496   1.016 1.003  3000
## deviance   252.842   4.119 246.071 250.101 252.252 254.985 262.679 1.003  1100
## 
## For each parameter, n.eff is a crude measure of effective sample size,
## and Rhat is the potential scale reduction factor (at convergence, Rhat=1).
## 
## DIC info (using the rule, pD = var(deviance)/2)
## pD = 8.5 and DIC = 261.3
## DIC is an estimate of expected predictive error (lower deviance is better).
```

```
autocorr.diag(as.mcmc(mod.nb.ind))
```

```
##                   alpha    beta_inter   beta_moist    beta_temp     deviance
## Lag 0       1.000000000  1.0000000000  1.000000000  1.000000000  1.000000000
## Lag 5000   -0.014767225  0.0125488624 -0.003084260 -0.025982340  0.042648959
## Lag 25000   0.001452361  0.0002969273 -0.027614268  0.012880415  0.012067779
## Lag 50000  -0.004017241 -0.0451538705 -0.008508741 -0.045194583 -0.022083927
## Lag 250000  0.010420354  0.0332968572  0.006848957 -0.006858615  0.003432637
##                      r      sigma_a
## Lag 0       1.00000000  1.000000000
## Lag 5000   -0.03857666 -0.003580251
## Lag 25000  -0.01865230 -0.014534395
## Lag 50000  -0.01580117 -0.004063320
## Lag 250000  0.01294030 -0.011517784
```

```
mod.pois.ind
```

```
## Inference for Bugs model at "C:/Users/mavecchi/AppData/Local/Temp/RtmpULkW5y/model2dc4969395b.txt", fit using jags,
##  3 chains, each with 1e+07 iterations (first 5e+06 discarded), n.thin = 5000
##  n.sims = 3000 iterations saved
##            mu.vect sd.vect    2.5%     25%     50%     75%   97.5%  Rhat n.eff
## alpha        4.066   0.198   3.650   3.950   4.075   4.185   4.458 1.001  3000
## beta_inter   0.601   0.295   0.028   0.426   0.604   0.777   1.205 1.001  3000
## beta_moist   0.014   0.203  -0.411  -0.104   0.013   0.135   0.419 1.001  3000
## beta_temp    0.176   0.202  -0.221   0.046   0.176   0.301   0.589 1.001  3000
## sigma_a      0.638   0.203   0.371   0.498   0.600   0.725   1.149 1.001  2400
## deviance   981.770   4.861 974.376 978.249 981.030 984.585 993.008 1.002  2000
## 
## For each parameter, n.eff is a crude measure of effective sample size,
## and Rhat is the potential scale reduction factor (at convergence, Rhat=1).
## 
## DIC info (using the rule, pD = var(deviance)/2)
## pD = 11.8 and DIC = 993.6
## DIC is an estimate of expected predictive error (lower deviance is better).
```

```
autocorr.diag(as.mcmc(mod.pois.ind))
```

```
##                    alpha   beta_inter   beta_moist    beta_temp    deviance
## Lag 0       1.000000e+00  1.000000000  1.000000000  1.000000000  1.00000000
## Lag 5000    8.629396e-03 -0.028172898  0.015800694  0.001446573  0.01273484
## Lag 25000   9.841429e-06 -0.034571779 -0.009168148 -0.006420854  0.03357700
## Lag 50000  -8.017653e-03  0.007161573 -0.010590466  0.016267915 -0.02056654
## Lag 250000 -2.024788e-02 -0.002724123 -0.020233584 -0.001811169  0.01008579
##                 sigma_a
## Lag 0       1.000000000
## Lag 5000   -0.002684358
## Lag 25000  -0.021328682
## Lag 50000   0.008158555
## Lag 250000  0.024442152
```

Despite the n.eff values are not ideal, the Rhat and autocorrelations looks fine, so I´ll keep those models.  
As the Negative Binomial model has a lower DIC the Poisson model will be discarded.

###Taxa

For the number of species I´ll try as well Negative Binomial and Poisson with a log link. Negative Binomial and Poisson are chosen because they´re discrete probability distributions that goes from 0 to +Inf, like the Taxa count index.

Let´s create the jags data for both the models:

```
data_jags=list(Nchamber=12,
                chamber=as.factor(tardigrades_alldata$Chamber),
                taxa=tardigrades_alldata$Taxa,
                temperature=as.numeric(scale(tardigrades_alldata$cat.avg)),
                moisture=as.numeric(scale(tardigrades_alldata$csm.avg)))
```

And run them

```
################################################
###### Taxa with Negative Binomial ######
################################################

mod_jags_nb <- function(){
  # Priors
  alpha ~ dunif(-1000,1000)# intercept
  beta_temp ~ dunif(-1000,1000)# beta for temperature
  beta_moist ~ dunif(-1000,1000)# beta for soil moisture
  beta_inter ~ dunif(-1000,1000)# beta for interaction
  sigma_a ~ dunif(0, 100) # standard deviation of random effect (variance between sites)
  tau_a <- 1 / (sigma_a * sigma_a) # convert to precision
  #r ~ dgamma(0.1 , 0.1) # prior for overdispersion parameter according to: http://doingbayesiandataanalysis.blogspot.com/2012/04/negative-binomial-reparameterization.html
  r ~ dunif(0,100) # prior for overdispersion parameter 
  
  # Random intercept for each chamber (random effect)
  for (j in 1:Nchamber){
    a[j] ~ dnorm(0, tau_a) 
  }
  
  # Likelihood:
  for (i in 1:length(taxa)){
    mu[i] <- exp(alpha + a[chamber[i]] + beta_temp * temperature[i] + beta_moist * moisture[i] + beta_inter * temperature[i] * moisture[i]) #predicted values with log link
    p[i] <- r / ( r + mu[i]) #reparametrization of negbin parameters according to: http://doingbayesiandataanalysis.blogspot.com/2012/04/negative-binomial-reparameterization.html
    taxa[i] ~ dnegbin( p[i] , r )
  }
}

mod_params_nb <- c("alpha", "beta_temp", "beta_moist", "beta_inter", "sigma_a","r")

mod.nb.taxa=jags(data=data_jags,parameters.to.save=mod_params_nb, model.file=mod_jags_nb,n.iter=10000000)

######################################
###### Taxa with Poisson ######
######################################

mod_jags_pois <- function(){
  # Priors
  alpha ~ dunif(-1000,1000)# intercept
  beta_temp ~ dunif(-1000,1000)# beta for temperature
  beta_moist ~ dunif(-1000,1000)# beta for soil moisture
  beta_inter ~ dunif(-1000,1000)# beta for interaction
  sigma_a ~ dunif(0, 100) # standard deviation of random effect (variance between sites)
  tau_a <- 1 / (sigma_a * sigma_a) # convert to precision
  
  # Random intercept for each chamber (random effect)
  for (j in 1:Nchamber){
    a[j] ~ dnorm(0, tau_a) 
  }
  
  # Likelihood:
  for (i in 1:length(taxa)){
    mu[i] <- exp(alpha + a[chamber[i]] + beta_temp * temperature[i] + beta_moist * moisture[i] + beta_inter * temperature[i] * moisture[i]) #predicted values with log link
    taxa[i] ~ dpois(mu[i])
  }
}

mod_params_pois <- c("alpha", "beta_temp", "beta_moist", "beta_inter", "sigma_a")


mod.pois.taxa=jags(data=data_jags,parameters.to.save=mod_params_pois, model.file=mod_jags_pois,n.iter=10000000)
```

Now check if the models converged and their DICS

```
mod.nb.taxa
```

```
## Inference for Bugs model at "C:/Users/mavecchi/AppData/Local/Temp/RtmpULkW5y/model2dc423049f2.txt", fit using jags,
##  3 chains, each with 1e+07 iterations (first 5e+06 discarded), n.thin = 5000
##  n.sims = 3000 iterations saved
##            mu.vect sd.vect   2.5%    25%    50%    75%   97.5%  Rhat n.eff
## alpha        1.433   0.119  1.199  1.356  1.432  1.511   1.663 1.001  2400
## beta_inter  -0.046   0.187 -0.414 -0.163 -0.047  0.074   0.319 1.001  3000
## beta_moist   0.091   0.121 -0.143  0.017  0.090  0.168   0.332 1.002  1400
## beta_temp    0.009   0.124 -0.232 -0.073  0.008  0.088   0.252 1.001  3000
## r           60.111  24.806 14.606 39.715 61.625 82.104  98.242 1.001  3000
## sigma_a      0.139   0.117  0.004  0.052  0.109  0.195   0.431 1.001  3000
## deviance    96.516   3.413 91.826 93.985 95.907 98.249 104.972 1.001  2300
## 
## For each parameter, n.eff is a crude measure of effective sample size,
## and Rhat is the potential scale reduction factor (at convergence, Rhat=1).
## 
## DIC info (using the rule, pD = var(deviance)/2)
## pD = 5.8 and DIC = 102.3
## DIC is an estimate of expected predictive error (lower deviance is better).
```

```
autocorr.diag(as.mcmc(mod.nb.taxa))
```

```
##                   alpha   beta_inter   beta_moist    beta_temp     deviance
## Lag 0       1.000000000  1.000000000  1.000000000  1.000000000  1.000000000
## Lag 5000   -0.004638291 -0.010981265 -0.030078559  0.002338742  0.003436071
## Lag 25000  -0.022438803  0.017238436  0.025007280  0.002927836  0.005166846
## Lag 50000   0.003943955 -0.022242206 -0.014525064  0.024374551  0.012150790
## Lag 250000 -0.011927857 -0.003771181  0.005630438 -0.032644266 -0.026915770
##                       r      sigma_a
## Lag 0       1.000000000  1.000000000
## Lag 5000    0.016382548 -0.006554441
## Lag 25000  -0.018939791 -0.012225687
## Lag 50000   0.002594722 -0.016964300
## Lag 250000  0.023431556 -0.025085635
```

```
mod.pois.taxa
```

```
## Inference for Bugs model at "C:/Users/mavecchi/AppData/Local/Temp/RtmpULkW5y/model2dc15de81a.txt", fit using jags,
##  3 chains, each with 1e+07 iterations (first 5e+06 discarded), n.thin = 5000
##  n.sims = 3000 iterations saved
##            mu.vect sd.vect   2.5%    25%    50%    75%   97.5%  Rhat n.eff
## alpha        1.431   0.112  1.205  1.358  1.431  1.506   1.652 1.001  3000
## beta_inter  -0.049   0.173 -0.391 -0.160 -0.045  0.066   0.285 1.002  1900
## beta_moist   0.089   0.114 -0.138  0.014  0.090  0.166   0.312 1.002  1800
## beta_temp    0.012   0.120 -0.220 -0.065  0.011  0.090   0.253 1.002  2700
## sigma_a      0.134   0.113  0.005  0.048  0.106  0.189   0.422 1.001  3000
## deviance    95.171   3.073 91.018 92.887 94.638 96.823 102.458 1.002  1100
## 
## For each parameter, n.eff is a crude measure of effective sample size,
## and Rhat is the potential scale reduction factor (at convergence, Rhat=1).
## 
## DIC info (using the rule, pD = var(deviance)/2)
## pD = 4.7 and DIC = 99.9
## DIC is an estimate of expected predictive error (lower deviance is better).
```

```
autocorr.diag(as.mcmc(mod.pois.taxa))
```

```
##                    alpha  beta_inter  beta_moist    beta_temp     deviance
## Lag 0       1.0000000000  1.00000000  1.00000000  1.000000000  1.000000000
## Lag 5000    0.0003648238  0.02701803 -0.01397509 -0.001664092  0.014988351
## Lag 25000  -0.0107868660  0.01219609 -0.02523087 -0.003975447  0.001527522
## Lag 50000  -0.0151653750 -0.02809005  0.01637304 -0.012277953  0.004931157
## Lag 250000  0.0095430451 -0.01844391 -0.01683274  0.007331678 -0.005914477
##                 sigma_a
## Lag 0       1.000000000
## Lag 5000   -0.023847736
## Lag 25000   0.002613071
## Lag 50000   0.035519340
## Lag 250000 -0.030064685
```

Despite the n.eff values are not ideal, the Rhat and autocorrelations looks fine, so I´ll keep those models.  
As the Poisson model has a lower DIC the Negative Binomial model will be discarded.

###Shannon

For the Shannon index I´ll use a Gamma family with a log link. Gamma is chosen because it´s a continuous probability distributions that goes from 0 to +Inf, like the Shannon index.

Let´s create the jags data:

```
data_jags=list(Nchamber=12,
                chamber=as.factor(tardigrades_alldata$Chamber),
            shannon=tardigrades_alldata$Shannon,
                temperature=as.numeric(scale(tardigrades_alldata$cat.avg)),
                moisture=as.numeric(scale(tardigrades_alldata$csm.avg)))
```

And run them

```
################################################
###### Shannon with Gamma ######
################################################

mod_jags <- function(){
  # Priors
  alpha ~ dunif(-1000,1000)# intercept
  beta_temp ~ dunif(-1000,1000)# beta for temperature
  beta_moist ~ dunif(-1000,1000)# beta for soil moisture
  beta_inter ~ dunif(-1000,1000)# beta for interaction
  sigma_a ~ dunif(0, 100) # standard deviation of random effect (variance between sites)
  tau_a <- 1 / (sigma_a * sigma_a) # convert to precision
  sigma ~ dunif(0, 100) # standard deviation of Gamma distribution
  
  # Random intercept for each chamber (random effect)
  for (j in 1:Nchamber){
    a[j] ~ dnorm(0, tau_a) 
  }
  
  # Likelihood:
  for (i in 1:length(shannon)){
    mu[i] <- exp(alpha + a[chamber[i]] + beta_temp * temperature[i] + beta_moist * moisture[i] + beta_inter * temperature[i] * moisture[i]) #predicted values with log link
    sh[i] <- pow(mu[i],2)/pow(sigma,2) # Shape parameter of Gamma distribution reparametrized according to : http://doingbayesiandataanalysis.blogspot.com/2012/08/gamma-likelihood-parameterized-by-mode.html
    ra[i] <- mu[i]/pow(sigma,2)        # Rate parameter of Gamma distribution reparametrized according to : http://doingbayesiandataanalysis.blogspot.com/2012/08/gamma-likelihood-parameterized-by-mode.html
    shannon[i] ~ dgamma(sh[i],ra[i])
  }
}

mod_params <- c("alpha", "beta_temp", "beta_moist", "beta_inter", "sigma_a","sigma")


mod.sha=jags(data=data_jags,parameters.to.save=mod_params, model.file=mod_jags,n.iter=10000000)
```

Now check if the model converged

```
mod.sha
```

```
## Inference for Bugs model at "C:/Users/mavecchi/AppData/Local/Temp/RtmpULkW5y/model2dc53a84bb9.txt", fit using jags,
##  3 chains, each with 1e+07 iterations (first 5e+06 discarded), n.thin = 5000
##  n.sims = 3000 iterations saved
##            mu.vect sd.vect   2.5%    25%    50%    75%  97.5%  Rhat n.eff
## alpha       -0.129   0.092 -0.320 -0.186 -0.126 -0.069  0.047 1.001  3000
## beta_inter  -0.144   0.130 -0.403 -0.228 -0.141 -0.059  0.106 1.001  2200
## beta_moist  -0.006   0.088 -0.192 -0.061 -0.002  0.052  0.158 1.001  3000
## beta_temp    0.023   0.086 -0.140 -0.031  0.022  0.077  0.194 1.002  1400
## sigma        0.334   0.062  0.240  0.291  0.324  0.367  0.476 1.001  3000
## sigma_a      0.103   0.086  0.004  0.039  0.082  0.145  0.323 1.001  3000
## deviance    13.340   4.113  7.432 10.336 12.654 15.617 23.422 1.001  3000
## 
## For each parameter, n.eff is a crude measure of effective sample size,
## and Rhat is the potential scale reduction factor (at convergence, Rhat=1).
## 
## DIC info (using the rule, pD = var(deviance)/2)
## pD = 8.5 and DIC = 21.8
## DIC is an estimate of expected predictive error (lower deviance is better).
```

```
autocorr.diag(as.mcmc(mod.sha))
```

```
##                   alpha   beta_inter   beta_moist    beta_temp     deviance
## Lag 0       1.000000000  1.000000000  1.000000000  1.000000000  1.000000000
## Lag 5000   -0.016050562  0.017341200 -0.030154644  0.002994695  0.037587432
## Lag 25000   0.028819835 -0.001059851  0.006897438  0.007824238  0.014777579
## Lag 50000  -0.006582956  0.014676913 -0.012529069  0.006018870 -0.004069875
## Lag 250000  0.016984825 -0.016123585  0.014423078 -0.006475205 -0.002864560
##                  sigma      sigma_a
## Lag 0      1.000000000  1.000000000
## Lag 5000   0.016867346  0.021740237
## Lag 25000  0.035198714 -0.002401006
## Lag 50000  0.003768026 -0.022707184
## Lag 250000 0.025873697 -0.023649826
```

### Adropion

For the Adropion individuals i´ll try two different error families: negative binomial and poisson. For both of them I´ll test with or without zero inflation as there are some replicates with no Adropion individuals. The link function is log

Let´s create the jags data:

```
data_jags=list(Nchamber=12,
               chamber=as.factor(tardigrades_alldata$Chamber),
               individuals=tardigrades_alldata$Adropion,
               temperature=as.numeric(scale(tardigrades_alldata$cat.avg)),
               moisture=as.numeric(scale(tardigrades_alldata$csm.avg)))
```

And run them:

```
################################################
###### Adropion with Negative Binomial     ######
################################################

mod_jags_nb <- function(){
  # Priors
  alpha ~ dunif(-1000,1000)# intercept
  beta_temp ~ dunif(-1000,1000)# beta for temperature
  beta_moist ~ dunif(-1000,1000)# beta for soil moisture
  beta_inter ~ dunif(-1000,1000)# beta for interaction
  sigma_a ~ dunif(0, 100) # standard deviation of random effect (variance between sites)
  tau_a <- 1 / (sigma_a * sigma_a) # convert to precision
  r ~ dgamma(0.1 , 0.1) # prior for overdispersion parameter according to: http://doingbayesiandataanalysis.blogspot.com/2012/04/negative-binomial-reparameterization.html
  
  # Random intercept for each chamber (random effect)
  for (j in 1:Nchamber){
    a[j] ~ dnorm(0, tau_a) 
  }
  
  # Likelihood:
  for (i in 1:length(individuals)){
    mu[i] <- exp(alpha + a[chamber[i]] + beta_temp * temperature[i] + beta_moist * moisture[i] + beta_inter * temperature[i] * moisture[i]) #predicted values with log link
    p[i] <- r / ( r + mu[i]) #reparametrization of negbin parameters according to: http://doingbayesiandataanalysis.blogspot.com/2012/04/negative-binomial-reparameterization.html
    individuals[i] ~ dnegbin( p[i] , r )
  }
}

mod_params_nb <- c("alpha", "beta_temp", "beta_moist", "beta_inter", "sigma_a","r")


mod.nb.adr=jags(data=data_jags,parameters.to.save=mod_params_nb, model.file=mod_jags_nb,n.iter=100000)

##################################
###### Adropion with Poisson ######
##################################

mod_jags_pois <- function(){
  # Priors
  alpha ~ dunif(-1000,1000)# intercept
  beta_temp ~ dunif(-1000,1000)# beta for temperature
  beta_moist ~ dunif(-1000,1000)# beta for soil moisture
  beta_inter ~ dunif(-1000,1000)# beta for interaction
  sigma_a ~ dunif(0, 100) # standard deviation of random effect (variance between sites)
  tau_a <- 1 / (sigma_a * sigma_a) # convert to precision
  
  # Random intercept for each chamber (random effect)
  for (j in 1:Nchamber){
    a[j] ~ dnorm(0, tau_a) 
  }
  
  # Likelihood:
  for (i in 1:length(individuals)){
    mu[i] <- exp(alpha + a[chamber[i]] + beta_temp * temperature[i] + beta_moist * moisture[i] + beta_inter * temperature[i] * moisture[i]) #predicted values with log link
    individuals[i] ~ dpois(mu[i])
  }
}

mod_params_pois <- c("alpha", "beta_temp", "beta_moist", "beta_inter", "sigma_a")


mod.pois.adr=jags(data=data_jags,parameters.to.save=mod_params_pois, model.file=mod_jags_pois,n.iter=100000)

####################################################################
###### Adropion with Negative Binomial with zero inflation     ######
####################################################################

mod_jags_nb <- function(){
  # Priors
  alpha ~ dunif(-1000,1000)# intercept
  beta_temp ~ dunif(-1000,1000)# beta for temperature
  beta_moist ~ dunif(-1000,1000)# beta for soil moisture
  beta_inter ~ dunif(-1000,1000)# beta for interaction
  sigma_a ~ dunif(0, 100) # standard deviation of random effect (variance between sites)
  tau_a <- 1 / (sigma_a * sigma_a) # convert to precision
  r ~ dgamma(0.1 , 0.1) # prior for overdispersion parameter according to: http://doingbayesiandataanalysis.blogspot.com/2012/04/negative-binomial-reparameterization.html
  psi ~ dunif(0, 1) # proportion of non-zeros
  psi_zeros <- 1-psi #proportion of zeros
  
  # Random intercept for each chamber (random effect)
  for (j in 1:Nchamber){
    a[j] ~ dnorm(0, tau_a) 
  }
  
  # Likelihood:
  for (i in 1:length(individuals)){
    mu[i] <- exp(alpha + a[chamber[i]] + beta_temp * temperature[i] + beta_moist * moisture[i] + beta_inter * temperature[i] * moisture[i]) #predicted values with log link
    
    z[i] ~ dbern(psi) #zero inflation
    mu_zeroinfl[i] <- mu[i]*z[i] + 0.00001*(1-z[i]) ## hack required for Rjags -- otherwise 'incompatible'-error
    
    p[i] <- r / ( r + mu_zeroinfl[i]) #reparametrization of negbin parameters according to: http://doingbayesiandataanalysis.blogspot.com/2012/04/negative-binomial-reparameterization.html
    individuals[i] ~ dnegbin( p[i] , r )
  }
}

mod_params_nb <- c("alpha", "beta_temp", "beta_moist", "beta_inter", "sigma_a","r","psi_zeros")

mod.nb.zero.adr=jags(data=data_jags,parameters.to.save=mod_params_nb, model.file=mod_jags_nb,n.iter=100000)

######################################################
###### Adropion with Poisson with zero inflation ######
######################################################

mod_jags_pois <- function(){
  # Priors
  alpha ~ dunif(-1000,1000)# intercept
  beta_temp ~ dunif(-1000,1000)# beta for temperature
  beta_moist ~ dunif(-1000,1000)# beta for soil moisture
  beta_inter ~ dunif(-1000,1000)# beta for interaction
  sigma_a ~ dunif(0, 100) # standard deviation of random effect (variance between sites)
  tau_a <- 1 / (sigma_a * sigma_a) # convert to precision
  psi ~ dunif(0, 1) # proportion of non-zeros
  psi_zeros <- 1-psi #proportion of zeros
  
  # Random intercept for each chamber (random effect)
  for (j in 1:Nchamber){
    a[j] ~ dnorm(0, tau_a) 
  }
  
  # Likelihood:
  for (i in 1:length(individuals)){
    mu[i] <- exp(alpha + a[chamber[i]] + beta_temp * temperature[i] + beta_moist * moisture[i] + beta_inter * temperature[i] * moisture[i]) #predicted values with log link
    
    z[i] ~ dbern(psi) #zero inflation
    mu_zeroinfl[i] <- mu[i]*z[i] + 0.00001*(1-z[i]) ## hack required for Rjags -- otherwise 'incompatible'-error
    
    individuals[i] ~ dpois(mu_zeroinfl[i])
  }
}

mod_params_pois <- c("alpha", "beta_temp", "beta_moist", "beta_inter", "sigma_a","psi_zeros")


mod.pois.zero.adr=jags(data=data_jags,parameters.to.save=mod_params_pois, model.file=mod_jags_pois,n.iter=100000)
```

Now check if the models converged

Negative Binomial

```
mod.nb.adr
```

```
## Inference for Bugs model at "C:/Users/mavecchi/AppData/Local/Temp/RtmpEHuhOm/model7f464b61af8.txt", fit using jags,
##  3 chains, each with 5e+07 iterations (first 2.5e+07 discarded), n.thin = 25000
##  n.sims = 3000 iterations saved
##            mu.vect sd.vect    2.5%     25%     50%     75%   97.5%  Rhat n.eff
## alpha        3.251   0.924   1.394   2.695   3.269   3.802   5.103 1.001  2100
## beta_inter  -0.593   1.683  -4.045  -1.588  -0.499   0.500   2.500 1.001  2800
## beta_moist   0.575   0.948  -1.104  -0.008   0.511   1.102   2.716 1.001  3000
## beta_temp    0.278   0.928  -1.558  -0.259   0.272   0.808   2.223 1.001  3000
## r            0.284   0.116   0.121   0.201   0.263   0.342   0.565 1.001  3000
## sigma_a      1.764   1.225   0.111   0.868   1.563   2.367   4.785 1.003  1900
## deviance   177.879   6.425 165.901 173.421 177.942 182.092 191.531 1.001  3000
## 
## For each parameter, n.eff is a crude measure of effective sample size,
## and Rhat is the potential scale reduction factor (at convergence, Rhat=1).
## 
## DIC info (using the rule, pD = var(deviance)/2)
## pD = 20.6 and DIC = 198.5
## DIC is an estimate of expected predictive error (lower deviance is better).
```

```
autocorr.diag(as.mcmc(mod.nb.adr))
```

```
##                    alpha   beta_inter   beta_moist    beta_temp      deviance
## Lag 0        1.000000000  1.000000000  1.000000000  1.000000000  1.0000000000
## Lag 25000    0.030193116  0.036406285 -0.009865744 -0.018132501 -0.0023324002
## Lag 125000   0.028403908  0.014504814 -0.010416461 -0.013376596  0.0255883787
## Lag 250000  -0.007257613 -0.002491055  0.004785907  0.011029282 -0.0035889148
## Lag 1250000 -0.006403734 -0.021257595 -0.014210202  0.003697794 -0.0004127245
##                        r      sigma_a
## Lag 0        1.000000000  1.000000000
## Lag 25000    0.012109772  0.022839594
## Lag 125000  -0.005030072 -0.007760627
## Lag 250000  -0.016213769 -0.006100017
## Lag 1250000 -0.015820500 -0.002816598
```

Poisson

```
mod.pois.adr
```

```
## Inference for Bugs model at "C:/Users/mavecchi/AppData/Local/Temp/RtmpEHuhOm/model7f46dab2ad2.txt", fit using jags,
##  3 chains, each with 5e+07 iterations (first 2.5e+07 discarded), n.thin = 25000
##  n.sims = 3000 iterations saved
##            mu.vect sd.vect    2.5%     25%     50%     75%   97.5%  Rhat n.eff
## alpha        2.163   0.890   0.384   1.666   2.187   2.685   3.847 1.002  2400
## beta_inter  -1.026   1.462  -4.268  -1.801  -0.925  -0.119   1.565 1.001  3000
## beta_moist   0.562   0.961  -1.162   0.001   0.513   1.087   2.554 1.003  1400
## beta_temp    0.273   0.921  -1.482  -0.271   0.233   0.795   2.160 1.003  3000
## sigma_a      2.739   1.047   1.461   2.047   2.505   3.172   5.419 1.002  2000
## deviance   700.005   4.828 692.534 696.507 699.355 702.907 710.885 1.001  3000
## 
## For each parameter, n.eff is a crude measure of effective sample size,
## and Rhat is the potential scale reduction factor (at convergence, Rhat=1).
## 
## DIC info (using the rule, pD = var(deviance)/2)
## pD = 11.7 and DIC = 711.7
## DIC is an estimate of expected predictive error (lower deviance is better).
```

```
autocorr.diag(as.mcmc(mod.pois.adr))
```

```
##                   alpha   beta_inter  beta_moist   beta_temp     deviance
## Lag 0        1.00000000  1.000000000  1.00000000  1.00000000  1.000000000
## Lag 25000   -0.01281938 -0.014970085 -0.02469612 -0.01062402  0.003990383
## Lag 125000   0.01783190 -0.006035115 -0.01833981  0.01634186 -0.005689591
## Lag 250000   0.01488922  0.013175744 -0.00827999  0.01893059 -0.009064581
## Lag 1250000  0.01316711  0.024363215  0.01322477  0.04689953 -0.008985661
##                  sigma_a
## Lag 0        1.000000000
## Lag 25000   -0.006601437
## Lag 125000   0.001632153
## Lag 250000  -0.003650062
## Lag 1250000  0.013264751
```

Zero-inflated negative binomial

```
mod.nb.zero.adr
```

```
## Inference for Bugs model at "C:/Users/mavecchi/AppData/Local/Temp/RtmpEHuhOm/model7f468ef2807.txt", fit using jags,
##  3 chains, each with 5e+07 iterations (first 2.5e+07 discarded), n.thin = 25000
##  n.sims = 3000 iterations saved
##            mu.vect sd.vect    2.5%     25%     50%     75%   97.5%  Rhat n.eff
## alpha        3.314   0.902   1.534   2.785   3.315   3.847   5.090 1.002  1100
## beta_inter  -0.162   1.753  -4.060  -1.181  -0.048   1.011   2.952 1.003  3000
## beta_moist   0.370   0.929  -1.386  -0.209   0.356   0.899   2.363 1.001  3000
## beta_temp    0.108   0.894  -1.733  -0.397   0.121   0.643   1.878 1.001  3000
## psi_zeros    0.151   0.105   0.007   0.068   0.134   0.217   0.392 1.001  3000
## r            0.437   0.253   0.149   0.267   0.369   0.538   1.103 1.001  3000
## sigma_a      1.724   1.192   0.102   0.874   1.534   2.314   4.667 1.006   400
## deviance   167.504  10.604 145.651 160.232 168.421 175.402 186.354 1.003   890
## 
## For each parameter, n.eff is a crude measure of effective sample size,
## and Rhat is the potential scale reduction factor (at convergence, Rhat=1).
## 
## DIC info (using the rule, pD = var(deviance)/2)
## pD = 56.1 and DIC = 223.6
## DIC is an estimate of expected predictive error (lower deviance is better).
```

```
autocorr.diag(as.mcmc(mod.nb.zero.adr))
```

```
##                   alpha   beta_inter   beta_moist  beta_temp      deviance
## Lag 0        1.00000000  1.000000000  1.000000000 1.00000000  1.0000000000
## Lag 25000   -0.01021861 -0.012406551 -0.021028126 0.03630726 -0.0009835214
## Lag 125000  -0.02291028 -0.014111046 -0.016386808 0.01473651  0.0138510249
## Lag 250000  -0.01249606 -0.009879171 -0.009740927 0.02322990 -0.0284322430
## Lag 1250000  0.01299666 -0.009218881 -0.030090560 0.02228459 -0.0049201858
##               psi_zeros            r    sigma_a
## Lag 0       1.000000000  1.000000000 1.00000000
## Lag 25000   0.018370360  0.012588682 0.05148501
## Lag 125000  0.004810960  0.003229711 0.01833161
## Lag 250000  0.005959803 -0.024678826 0.00496689
## Lag 1250000 0.015458931 -0.014912428 0.01011750
```

Zero-inflated poisson

```
mod.pois.zero.adr
```

```
## Inference for Bugs model at "C:/Users/mavecchi/AppData/Local/Temp/RtmpEHuhOm/model7f45ae87e0c.txt", fit using jags,
##  3 chains, each with 5e+07 iterations (first 2.5e+07 discarded), n.thin = 25000
##  n.sims = 3000 iterations saved
##            mu.vect sd.vect    2.5%     25%     50%     75%   97.5%  Rhat n.eff
## alpha        2.803   0.796   1.145   2.354   2.847   3.306   4.276 1.001  3000
## beta_inter   0.078   1.628  -3.392  -0.886   0.208   1.100   3.045 1.001  3000
## beta_moist   0.006   0.887  -1.629  -0.553  -0.040   0.518   1.931 1.002  3000
## beta_temp   -0.153   0.851  -1.768  -0.651  -0.174   0.317   1.611 1.003  1300
## psi_zeros    0.322   0.099   0.145   0.252   0.318   0.386   0.521 1.001  2100
## sigma_a      2.347   0.972   1.190   1.707   2.114   2.699   4.790 1.001  3000
## deviance   413.820   5.094 406.035 410.122 413.148 416.774 425.825 1.001  3000
## 
## For each parameter, n.eff is a crude measure of effective sample size,
## and Rhat is the potential scale reduction factor (at convergence, Rhat=1).
## 
## DIC info (using the rule, pD = var(deviance)/2)
## pD = 13.0 and DIC = 426.8
## DIC is an estimate of expected predictive error (lower deviance is better).
```

```
autocorr.diag(as.mcmc(mod.pois.zero.adr))
```

```
##                   alpha   beta_inter    beta_moist    beta_temp     deviance
## Lag 0        1.00000000  1.000000000  1.0000000000  1.000000000  1.000000000
## Lag 25000    0.02535302 -0.017266251 -0.0007903152  0.018756442  0.032076313
## Lag 125000   0.00331940  0.003263185 -0.0257317565 -0.004787209 -0.006803058
## Lag 250000  -0.01887991  0.054897654  0.0082557358  0.005722309 -0.002818889
## Lag 1250000  0.03993722 -0.003282423 -0.0414334387  0.015102579  0.015600677
##               psi_zeros       sigma_a
## Lag 0        1.00000000  1.0000000000
## Lag 25000   -0.01944774 -0.0302347245
## Lag 125000  -0.01280895 -0.0007164413
## Lag 250000   0.02337970  0.0120195304
## Lag 1250000 -0.01365271 -0.0060471734
```

As the Negative binomial model has a lower DIC, all the other models will be discarded.

### Diphascon

For the Diphascon individuals i´ll try two different error families: negative binomial and poisson. As there are no zero´s I won´t test the zero inflated models. The link function is log

Let´s create the jags data:

```
#Data
data_jags=list(Nchamber=12,
               chamber=as.factor(tardigrades_alldata$Chamber),
               individuals=tardigrades_alldata$Diphascon,
               temperature=as.numeric(scale(tardigrades_alldata$cat.avg)),
               moisture=as.numeric(scale(tardigrades_alldata$csm.avg)))
```

And run them:

```
################################################
###### Diphascon with Negative Binomial   ######
################################################

mod_jags_nb <- function(){
  # Priors
  alpha ~ dunif(-1000,1000)# intercept
  beta_temp ~ dunif(-1000,1000)# beta for temperature
  beta_moist ~ dunif(-1000,1000)# beta for soil moisture
  beta_inter ~ dunif(-1000,1000)# beta for interaction
  sigma_a ~ dunif(0, 100) # standard deviation of random effect (variance between sites)
  tau_a <- 1 / (sigma_a * sigma_a) # convert to precision
  r ~ dgamma(0.1 , 0.1) # prior for overdispersion parameter according to: http://doingbayesiandataanalysis.blogspot.com/2012/04/negative-binomial-reparameterization.html
  
  # Random intercept for each chamber (random effect)
  for (j in 1:Nchamber){
    a[j] ~ dnorm(0, tau_a) 
  }
  
  # Likelihood:
  for (i in 1:length(individuals)){
    mu[i] <- exp(alpha + a[chamber[i]] + beta_temp * temperature[i] + beta_moist * moisture[i] + beta_inter * temperature[i] * moisture[i]) #predicted values with log link
    p[i] <- r / ( r + mu[i]) #reparametrization of negbin parameters according to: http://doingbayesiandataanalysis.blogspot.com/2012/04/negative-binomial-reparameterization.html
    individuals[i] ~ dnegbin( p[i] , r )
  }
}

mod_params_nb <- c("alpha", "beta_temp", "beta_moist", "beta_inter", "sigma_a","r")


mod.nb.dip=jags(data=data_jags,parameters.to.save=mod_params_nb, model.file=mod_jags_nb,n.iter=100000)

##################################
###### Diphascon with Poisson ######
##################################

mod_jags_pois <- function(){
  # Priors
  alpha ~ dunif(-1000,1000)# intercept
  beta_temp ~ dunif(-1000,1000)# beta for temperature
  beta_moist ~ dunif(-1000,1000)# beta for soil moisture
  beta_inter ~ dunif(-1000,1000)# beta for interaction
  sigma_a ~ dunif(0, 100) # standard deviation of random effect (variance between sites)
  tau_a <- 1 / (sigma_a * sigma_a) # convert to precision
  
  # Random intercept for each chamber (random effect)
  for (j in 1:Nchamber){
    a[j] ~ dnorm(0, tau_a) 
  }
  
  # Likelihood:
  for (i in 1:length(individuals)){
    mu[i] <- exp(alpha + a[chamber[i]] + beta_temp * temperature[i] + beta_moist * moisture[i] + beta_inter * temperature[i] * moisture[i]) #predicted values with log link
    individuals[i] ~ dpois(mu[i])
  }
}

mod_params_pois <- c("alpha", "beta_temp", "beta_moist", "beta_inter", "sigma_a")


mod.pois.dip=jags(data=data_jags,parameters.to.save=mod_params_pois, model.file=mod_jags_pois,n.iter=100000)
```

Now check if the models converged

Negative binomial

```
mod.nb.dip
```

```
## Inference for Bugs model at "C:/Users/mavecchi/AppData/Local/Temp/RtmpEHuhOm/model7f419f17a05.txt", fit using jags,
##  3 chains, each with 5e+07 iterations (first 2.5e+07 discarded), n.thin = 25000
##  n.sims = 3000 iterations saved
##            mu.vect sd.vect    2.5%     25%     50%     75%   97.5%  Rhat n.eff
## alpha        3.329   0.203   2.940   3.200   3.317   3.459   3.749 1.001  3000
## beta_inter   0.624   0.309   0.006   0.421   0.626   0.824   1.241 1.002  1500
## beta_moist  -0.062   0.207  -0.463  -0.194  -0.066   0.071   0.340 1.004  1200
## beta_temp    0.126   0.216  -0.323  -0.006   0.135   0.267   0.529 1.001  3000
## r            1.841   0.581   0.900   1.421   1.769   2.178   3.138 1.001  3000
## sigma_a      0.285   0.239   0.010   0.105   0.232   0.393   0.894 1.001  2100
## deviance   207.970   4.100 201.381 205.139 207.314 210.117 217.879 1.002  1500
## 
## For each parameter, n.eff is a crude measure of effective sample size,
## and Rhat is the potential scale reduction factor (at convergence, Rhat=1).
## 
## DIC info (using the rule, pD = var(deviance)/2)
## pD = 8.4 and DIC = 216.4
## DIC is an estimate of expected predictive error (lower deviance is better).
```

```
autocorr.diag(as.mcmc(mod.nb.dip))
```

```
##                    alpha   beta_inter   beta_moist     beta_temp    deviance
## Lag 0        1.000000000  1.000000000  1.000000000  1.0000000000  1.00000000
## Lag 25000   -0.002286821 -0.013726903  0.008782374  0.0001696699  0.02616636
## Lag 125000   0.023512850 -0.016000386 -0.025359166 -0.0391206511 -0.01172011
## Lag 250000   0.021146750 -0.006171965 -0.012506548 -0.0034525491 -0.02636889
## Lag 1250000  0.027742028  0.001697610 -0.006620379 -0.0070569150  0.03145715
##                        r      sigma_a
## Lag 0        1.000000000  1.000000000
## Lag 25000    0.026447211  0.016240886
## Lag 125000  -0.012530974  0.016286187
## Lag 250000  -0.002461104 -0.023827484
## Lag 1250000  0.009395300 -0.006229484
```

Poisson

```
mod.pois.dip
```

```
## Inference for Bugs model at "C:/Users/mavecchi/AppData/Local/Temp/RtmpEHuhOm/model7f451140b1.txt", fit using jags,
##  3 chains, each with 5e+07 iterations (first 2.5e+07 discarded), n.thin = 25000
##  n.sims = 3000 iterations saved
##            mu.vect sd.vect    2.5%     25%     50%     75%   97.5%  Rhat n.eff
## alpha        3.225   0.152   2.923   3.129   3.225   3.317   3.535 1.001  3000
## beta_inter   0.623   0.245   0.123   0.474   0.626   0.772   1.108 1.001  3000
## beta_moist  -0.074   0.158  -0.395  -0.171  -0.072   0.021   0.235 1.001  3000
## beta_temp    0.132   0.168  -0.183   0.033   0.130   0.230   0.472 1.001  3000
## sigma_a      0.483   0.167   0.260   0.371   0.451   0.557   0.902 1.001  3000
## deviance   440.870   4.864 433.271 437.370 440.260 443.717 452.070 1.001  3000
## 
## For each parameter, n.eff is a crude measure of effective sample size,
## and Rhat is the potential scale reduction factor (at convergence, Rhat=1).
## 
## DIC info (using the rule, pD = var(deviance)/2)
## pD = 11.8 and DIC = 452.7
## DIC is an estimate of expected predictive error (lower deviance is better).
```

```
autocorr.diag(as.mcmc(mod.pois.dip))
```

```
##                    alpha  beta_inter   beta_moist    beta_temp     deviance
## Lag 0        1.000000000  1.00000000  1.000000000  1.000000000 1.0000000000
## Lag 25000    0.013593446  0.00617901  0.025706572  0.035584250 0.0003355508
## Lag 125000   0.004907186  0.01166954 -0.001774369  0.004364737 0.0111869301
## Lag 250000  -0.011836973 -0.01919062  0.047237724  0.006964643 0.0268277669
## Lag 1250000 -0.016395911  0.02289012 -0.011809488 -0.015236543 0.0101698678
##                   sigma_a
## Lag 0        1.0000000000
## Lag 25000   -0.0024638495
## Lag 125000  -0.0104135087
## Lag 250000   0.0057345429
## Lag 1250000 -0.0003972702
```

As the Negative binomial model has a lower DIC, the poisson model will be discarded.

### Mesobiotus

For the Mesobiotus individuals i´ll try two different error families: negative binomial and poisson. As there are no zero´s I won´t test the zero inflated models. The link function is log

Let´s create the jags data:

```
#Data
data_jags=list(Nchamber=12,
               chamber=as.factor(tardigrades_alldata$Chamber),
               individuals=tardigrades_alldata$Mesobiotus,
               temperature=as.numeric(scale(tardigrades_alldata$cat.avg)),
               moisture=as.numeric(scale(tardigrades_alldata$csm.avg)))
```

And run them:

```
################################################
###### Mesobiotus with Negative Binomial     ######
################################################

mod_jags_nb <- function(){
  # Priors
  alpha ~ dunif(-1000,1000)# intercept
  beta_temp ~ dunif(-1000,1000)# beta for temperature
  beta_moist ~ dunif(-1000,1000)# beta for soil moisture
  beta_inter ~ dunif(-1000,1000)# beta for interaction
  sigma_a ~ dunif(0, 100) # standard deviation of random effect (variance between sites)
  tau_a <- 1 / (sigma_a * sigma_a) # convert to precision
  r ~ dgamma(0.1 , 0.1) # prior for overdispersion parameter according to: http://doingbayesiandataanalysis.blogspot.com/2012/04/negative-binomial-reparameterization.html
  
  # Random intercept for each chamber (random effect)
  for (j in 1:Nchamber){
    a[j] ~ dnorm(0, tau_a) 
  }
  
  # Likelihood:
  for (i in 1:length(individuals)){
    mu[i] <- exp(alpha + a[chamber[i]] + beta_temp * temperature[i] + beta_moist * moisture[i] + beta_inter * temperature[i] * moisture[i]) #predicted values with log link
    p[i] <- r / ( r + mu[i]) #reparametrization of negbin parameters according to: http://doingbayesiandataanalysis.blogspot.com/2012/04/negative-binomial-reparameterization.html
    individuals[i] ~ dnegbin( p[i] , r )
  }
}

mod_params_nb <- c("alpha", "beta_temp", "beta_moist", "beta_inter", "sigma_a","r")


mod.nb.mes=jags(data=data_jags,parameters.to.save=mod_params_nb, model.file=mod_jags_nb,n.iter=100000)

##################################
###### Mesobiotus with Poisson ######
##################################

mod_jags_pois <- function(){
  # Priors
  alpha ~ dunif(-1000,1000)# intercept
  beta_temp ~ dunif(-1000,1000)# beta for temperature
  beta_moist ~ dunif(-1000,1000)# beta for soil moisture
  beta_inter ~ dunif(-1000,1000)# beta for interaction
  sigma_a ~ dunif(0, 100) # standard deviation of random effect (variance between sites)
  tau_a <- 1 / (sigma_a * sigma_a) # convert to precision
  
  # Random intercept for each chamber (random effect)
  for (j in 1:Nchamber){
    a[j] ~ dnorm(0, tau_a) 
  }
  
  # Likelihood:
  for (i in 1:length(individuals)){
    mu[i] <- exp(alpha + a[chamber[i]] + beta_temp * temperature[i] + beta_moist * moisture[i] + beta_inter * temperature[i] * moisture[i]) #predicted values with log link
    individuals[i] ~ dpois(mu[i])
  }
}

mod_params_pois <- c("alpha", "beta_temp", "beta_moist", "beta_inter", "sigma_a")


mod.pois.mes=jags(data=data_jags,parameters.to.save=mod_params_pois, model.file=mod_jags_pois,n.iter=100000)
```

Now check if the models converged

Negative binomial

```
mod.nb.mes
```

```
## Inference for Bugs model at "C:/Users/mavecchi/AppData/Local/Temp/RtmpEHuhOm/model7f4a3167d0.txt", fit using jags,
##  3 chains, each with 5e+07 iterations (first 2.5e+07 discarded), n.thin = 25000
##  n.sims = 3000 iterations saved
##            mu.vect sd.vect    2.5%     25%     50%     75%   97.5%  Rhat n.eff
## alpha        2.226   0.246   1.741   2.069   2.225   2.381   2.723 1.002  1600
## beta_inter   0.620   0.374  -0.152   0.388   0.621   0.844   1.398 1.002  1900
## beta_moist  -0.385   0.269  -0.909  -0.552  -0.382  -0.217   0.152 1.001  3000
## beta_temp    0.148   0.250  -0.343  -0.006   0.152   0.300   0.653 1.002  1200
## r            1.567   0.590   0.731   1.142   1.481   1.860   3.011 1.002  2000
## sigma_a      0.372   0.302   0.017   0.150   0.299   0.514   1.115 1.001  2900
## deviance   159.695   4.329 152.165 156.810 159.237 162.190 169.427 1.003   960
## 
## For each parameter, n.eff is a crude measure of effective sample size,
## and Rhat is the potential scale reduction factor (at convergence, Rhat=1).
## 
## DIC info (using the rule, pD = var(deviance)/2)
## pD = 9.4 and DIC = 169.1
## DIC is an estimate of expected predictive error (lower deviance is better).
```

```
autocorr.diag(as.mcmc(mod.nb.mes))
```

```
##                    alpha   beta_inter    beta_moist   beta_temp     deviance
## Lag 0        1.000000000  1.000000000  1.0000000000  1.00000000  1.000000000
## Lag 25000    0.010519374  0.011601140  0.0004645878 -0.02530334  0.003893440
## Lag 125000   0.008066215  0.009832494  0.0212129314  0.02363947 -0.002122805
## Lag 250000  -0.003879319  0.011225228 -0.0123782009  0.02322150 -0.000743745
## Lag 1250000  0.006037989 -0.001639652  0.0246524278  0.01321173 -0.022847162
##                         r      sigma_a
## Lag 0        1.0000000000  1.000000000
## Lag 25000   -0.0007432245  0.017174740
## Lag 125000   0.0147585545  0.001929076
## Lag 250000   0.0142762694  0.038826976
## Lag 1250000 -0.0035028515 -0.015823415
```

Poisson

```
mod.pois.mes
```

```
## Inference for Bugs model at "C:/Users/mavecchi/AppData/Local/Temp/RtmpEHuhOm/model7f43eba4ad6.txt", fit using jags,
##  3 chains, each with 5e+07 iterations (first 2.5e+07 discarded), n.thin = 25000
##  n.sims = 3000 iterations saved
##            mu.vect sd.vect    2.5%     25%     50%     75%   97.5%  Rhat n.eff
## alpha        2.089   0.229   1.613   1.954   2.096   2.227   2.532 1.001  2100
## beta_inter   0.614   0.335  -0.046   0.410   0.601   0.812   1.313 1.004   530
## beta_moist  -0.375   0.232  -0.848  -0.514  -0.368  -0.232   0.066 1.001  2900
## beta_temp    0.150   0.228  -0.313   0.018   0.155   0.282   0.605 1.001  3000
## sigma_a      0.662   0.258   0.314   0.485   0.610   0.784   1.300 1.001  2300
## deviance   207.187   4.995 199.354 203.491 206.575 210.271 218.584 1.001  3000
## 
## For each parameter, n.eff is a crude measure of effective sample size,
## and Rhat is the potential scale reduction factor (at convergence, Rhat=1).
## 
## DIC info (using the rule, pD = var(deviance)/2)
## pD = 12.5 and DIC = 219.7
## DIC is an estimate of expected predictive error (lower deviance is better).
```

```
autocorr.diag(as.mcmc(mod.pois.mes))
```

```
##                    alpha   beta_inter   beta_moist    beta_temp      deviance
## Lag 0        1.000000000  1.000000000  1.000000000  1.000000000  1.0000000000
## Lag 25000   -0.013246586 -0.016167110  0.004388367  0.024306507  0.0004417025
## Lag 125000  -0.022223196  0.012955615  0.006500632  0.003889140  0.0132569500
## Lag 250000  -0.012090912 -0.001388656  0.007467265 -0.007632572  0.0117399732
## Lag 1250000 -0.004434223  0.001219155 -0.011449340  0.003689803 -0.0367623755
##                  sigma_a
## Lag 0        1.000000000
## Lag 25000   -0.013213110
## Lag 125000   0.028118244
## Lag 250000   0.004249039
## Lag 1250000 -0.013170938
```

As the Negative binomial model has a lower DIC, the poisson model will be discarded.

##Plotting the estimates

```
inds=data.frame(do.call(rbind,as.mcmc(mod.nb.ind)))[,2:4]
inds=gather(inds,"predictor","value")
inds$response=rep("Number of individuals",nrow(inds))

taxa=data.frame(do.call(rbind,as.mcmc(mod.pois.taxa)))[,2:4]
taxa=gather(taxa,"predictor","value")
taxa$response=rep("Number of taxa",nrow(taxa))

shan=data.frame(do.call(rbind,as.mcmc(mod.sha)))[,2:4]
shan=gather(shan,"predictor","value")
shan$response=rep("Shannon index",nrow(shan))

adr=data.frame(do.call(rbind,as.mcmc(mod.nb.adr)))[,2:4]
adr=gather(adr,"predictor","value")
adr$response=rep("Adropion",nrow(adr))

mes=data.frame(do.call(rbind,as.mcmc(mod.nb.mes)))[,2:4]
mes=gather(mes,"predictor","value")
mes$response=rep("Mesobiotus",nrow(mes))

dip=data.frame(do.call(rbind,as.mcmc(mod.nb.dip)))[,2:4]
dip=gather(dip,"predictor","value")
dip$response=rep("Diphascon",nrow(dip))

toplot=rbind(inds,taxa,shan,adr,mes,dip)

toplot$response=factor(toplot$response,levels=c("Number of individuals","Number of taxa","Shannon index","Adropion","Diphascon","Mesobiotus"))

p=ggplot(toplot)+
  theme_bw() +
  theme(panel.grid.major.x=element_blank(),
        panel.grid.minor.x=element_blank(),
        legend.position = "none",
        strip.background=element_blank(),
        strip.text=element_text(face="bold",size=10),
        plot.title = element_text(hjust = 0.5),
        axis.text.y = element_text())+
  geom_vline(xintercept = 0, col="red",alpha=0.5)+
  stat_density_ridges(aes(x=value,y=predictor,fill=factor(..quantile..)),
                      quantile_lines=T,
                      alpha=0.5,
                      panel_scaling=T,
                      scale=0.9,
                      geom = "density_ridges_gradient", 
                      calc_ecdf = TRUE, 
                      quantiles = c(0.025, 0.975)) +
  scale_fill_manual(name = "Probability", 
                    values = c("#CCCCCCA0", "#73E6FFA0", "#CCCCCCA0"),
                    labels = c("(0, 0.025]", "(0.025, 0.975]", "(0.975, 1]"))+
  scale_y_discrete(labels=c("beta_temp" = "Air temperature", "beta_moist" = "Soil moisture",
                              "beta_inter" = "Air temperature * Soil moisture"))+
  labs(x="Estimated value", y="Predictors",title="Models parameters estimates")+
  facet_wrap(.~response,scales="free_x")
p
```

```
## Picking joint bandwidth of 0.0489
```

```
## Picking joint bandwidth of 0.0241
```

```
## Picking joint bandwidth of 0.0176
```

```
## Picking joint bandwidth of 0.193
```

```
## Picking joint bandwidth of 0.0425
```

```
## Picking joint bandwidth of 0.0495
```

In all the parameters estimates for all the response variables the 0 (red line) falls inside the 95% of the estimated values.  
**No one of the tested predictor is different from zero.**

##Bayesian p-values calculation

```
# Individuals
inds=data.frame(do.call(rbind,as.mcmc(mod.nb.ind)))[,2:4]

pvals_inds = pd_to_p(p_direction(inds)$pd)
names(pvals_inds) = colnames(inds)

es_inds = colMedians(as.matrix((inds/sd(tardigrades_alldata$Individuals))))
names(es_inds) = colnames(inds)


# Taxa
taxa=data.frame(do.call(rbind,as.mcmc(mod.pois.taxa)))[,2:4]

pvals_taxa = pd_to_p(p_direction(taxa)$pd)
names(pvals_taxa) = colnames(taxa)

es_taxa = colMedians(as.matrix((taxa/sd(tardigrades_alldata$Taxa))))
names(es_taxa) = colnames(taxa)

#Shannon
shan=data.frame(do.call(rbind,as.mcmc(mod.sha)))[,2:4]

pvals_shan = pd_to_p(p_direction(shan)$pd)
names(pvals_shan) = colnames(shan)

es_shan = colMedians(as.matrix((shan/sd(tardigrades_alldata$Shannon))))
names(es_shan) = colnames(shan)

# Adropion
adr=data.frame(do.call(rbind,as.mcmc(mod.nb.adr)))[,2:4]

pvals_adr = pd_to_p(p_direction(adr)$pd)
names(pvals_adr) = colnames(adr)

es_adr = colMedians(as.matrix((adr/sd(tardigrades_alldata$Adropion))))
names(es_adr) = colnames(adr)

# Mesobiotus
mes=data.frame(do.call(rbind,as.mcmc(mod.nb.mes)))[,2:4]

pvals_mes = pd_to_p(p_direction(mes)$pd)
names(pvals_mes) = colnames(mes)

es_mes = colMedians(as.matrix((mes/sd(tardigrades_alldata$Mesobiotus))))
names(es_mes) = colnames(mes)

#Diphascon
dip=data.frame(do.call(rbind,as.mcmc(mod.nb.dip)))[,2:4]

pvals_dip = pd_to_p(p_direction(dip)$pd)
names(pvals_dip) = colnames(dip)

es_dip = colMedians(as.matrix((dip/sd(tardigrades_alldata$Diphascon))))
names(es_dip) = colnames(dip)


# Put them together

pvals_dataframe = data.frame(rbind(pvals_inds,
                                   pvals_taxa,
                                   pvals_shan,
                                   pvals_adr,
                                   pvals_mes,
                                   pvals_dip))

pvals_dataframe = pvals_dataframe[,c(3,2,1)]
write.table(pvals_dataframe,file="pvals.txt")


es_dataframe = data.frame(rbind(es_inds,
                                  es_taxa,
                                  es_shan,
                                  es_adr,
                                  es_mes,
                                  es_dip))

es_dataframe = es_dataframe[,c(3,2,1)]
write.table(es_dataframe,file="es.txt")
```

##Appendix 1: worm and density plots of models

```
toplot=data.frame(do.call(rbind,as.mcmc(mod.nb.ind)))
toplot$chain=c(rep("A",1000),rep("B",1000),rep("C",1000))
toplot$timepoint=c(1:1000,1:1000,1:1000)

toplot=gather(toplot,"parameter","value",1:7)

p_worm=ggplot(toplot)+
  geom_line(aes(x=timepoint,y=value,col=chain))+
  facet_grid(parameter~.,scales="free")+
  theme_bw()+
  ggtitle("Individuals - Negative Binomial")
p_dens=ggplot(toplot)+
  geom_density(aes(x=value,col=chain))+
  facet_wrap(parameter~.,scales="free")+
  theme_bw()+ggtitle("Individuals - Negative Binomial")
p_worm
```

```
p_dens
```

```
toplot=data.frame(do.call(rbind,as.mcmc(mod.pois.ind)))
toplot$chain=c(rep("A",1000),rep("B",1000),rep("C",1000))
toplot$timepoint=c(1:1000,1:1000,1:1000)

toplot=gather(toplot,"parameter","value",1:6)

p_worm=ggplot(toplot)+
  geom_line(aes(x=timepoint,y=value,col=chain))+
  facet_grid(parameter~.,scales="free")+
  theme_bw()+
  ggtitle("Individuals - Poisson")
p_dens=ggplot(toplot)+
  geom_density(aes(x=value,col=chain))+
  facet_wrap(parameter~.,scales="free")+
  theme_bw()+ggtitle("Individuals - Poisson")
p_worm
```

```
p_dens
```

```
toplot=data.frame(do.call(rbind,as.mcmc(mod.nb.taxa)))
toplot$chain=c(rep("A",1000),rep("B",1000),rep("C",1000))
toplot$timepoint=c(1:1000,1:1000,1:1000)

toplot=gather(toplot,"parameter","value",1:7)

p_worm=ggplot(toplot)+
  geom_line(aes(x=timepoint,y=value,col=chain))+
  facet_grid(parameter~.,scales="free")+
  theme_bw()+
  ggtitle("Taxa - Negative Binomial")
p_dens=ggplot(toplot)+
  geom_density(aes(x=value,col=chain))+
  facet_wrap(parameter~.,scales="free")+
  theme_bw()+ggtitle("Taxa - Negative Binomial")
p_worm
```

```
p_dens
```

```
toplot=data.frame(do.call(rbind,as.mcmc(mod.pois.taxa)))
toplot$chain=c(rep("A",1000),rep("B",1000),rep("C",1000))
toplot$timepoint=c(1:1000,1:1000,1:1000)

toplot=gather(toplot,"parameter","value",1:6)

p_worm=ggplot(toplot)+
  geom_line(aes(x=timepoint,y=value,col=chain))+
  facet_grid(parameter~.,scales="free")+
  theme_bw()+
  ggtitle("Taxa - Poisson")
p_dens=ggplot(toplot)+
  geom_density(aes(x=value,col=chain))+
  facet_wrap(parameter~.,scales="free")+
  theme_bw()+ggtitle("Taxa - Poisson")
p_worm
```

```
p_dens
```

```
toplot=data.frame(do.call(rbind,as.mcmc(mod.sha)))
toplot$chain=c(rep("A",1000),rep("B",1000),rep("C",1000))
toplot$timepoint=c(1:1000,1:1000,1:1000)

toplot=gather(toplot,"parameter","value",1:7)

p_worm=ggplot(toplot)+
  geom_line(aes(x=timepoint,y=value,col=chain))+
  facet_grid(parameter~.,scales="free")+
  theme_bw()+
  ggtitle("Shannon- Gamma")
p_dens=ggplot(toplot)+
  geom_density(aes(x=value,col=chain))+
  facet_wrap(parameter~.,scales="free")+
  theme_bw()+ggtitle("Shannon- Gamma")
p_worm
```

```
p_dens
```

```
toplot=data.frame(do.call(rbind,as.mcmc(mod.nb.adr)))
toplot$chain=c(rep("A",1000),rep("B",1000),rep("C",1000))
toplot$timepoint=c(1:1000,1:1000,1:1000)

toplot=gather(toplot,"parameter","value",1:7)

p_worm=ggplot(toplot)+
  geom_line(aes(x=timepoint,y=value,col=chain))+
  facet_grid(parameter~.,scales="free")+
  theme_bw()+
  ggtitle("Adropion - Negative Binomial")
p_dens=ggplot(toplot)+
  geom_density(aes(x=value,col=chain))+
  facet_wrap(parameter~.,scales="free")+
  theme_bw()+ggtitle("Adropion - Negative Binomial")
p_worm
```

```
p_dens
```

```
toplot=data.frame(do.call(rbind,as.mcmc(mod.nb.zero.adr)))
toplot$chain=c(rep("A",1000),rep("B",1000),rep("C",1000))
toplot$timepoint=c(1:1000,1:1000,1:1000)

toplot=gather(toplot,"parameter","value",1:8)

p_worm=ggplot(toplot)+
  geom_line(aes(x=timepoint,y=value,col=chain))+
  facet_grid(parameter~.,scales="free")+
  theme_bw()+
  ggtitle("Adropion - zero inflated Negative Binomial")
p_dens=ggplot(toplot)+
  geom_density(aes(x=value,col=chain))+
  facet_wrap(parameter~.,scales="free")+
  theme_bw()+ggtitle("Adropion - zero inflated Negative Binomial")
p_worm
```

```
p_dens
```

```
toplot=data.frame(do.call(rbind,as.mcmc(mod.pois.adr)))
toplot$chain=c(rep("A",1000),rep("B",1000),rep("C",1000))
toplot$timepoint=c(1:1000,1:1000,1:1000)

toplot=gather(toplot,"parameter","value",1:6)

p_worm=ggplot(toplot)+
  geom_line(aes(x=timepoint,y=value,col=chain))+
  facet_grid(parameter~.,scales="free")+
  theme_bw()+
  ggtitle("Adropion - Poisson")
p_dens=ggplot(toplot)+
  geom_density(aes(x=value,col=chain))+
  facet_wrap(parameter~.,scales="free")+
  theme_bw()+ggtitle("Adropion - Poisson")
p_worm
```

```
p_dens
```

```
toplot=data.frame(do.call(rbind,as.mcmc(mod.pois.zero.adr)))
toplot$chain=c(rep("A",1000),rep("B",1000),rep("C",1000))
toplot$timepoint=c(1:1000,1:1000,1:1000)

toplot=gather(toplot,"parameter","value",1:7)

p_worm=ggplot(toplot)+
  geom_line(aes(x=timepoint,y=value,col=chain))+
  facet_grid(parameter~.,scales="free")+
  theme_bw()+
  ggtitle("Adropion - zero inflated Poisson")
p_dens=ggplot(toplot)+
  geom_density(aes(x=value,col=chain))+
  facet_wrap(parameter~.,scales="free")+
  theme_bw()+ggtitle("Adropion - zero inflated Poisson")
p_worm
```

```
p_dens
```

```
toplot=data.frame(do.call(rbind,as.mcmc(mod.nb.mes)))
toplot$chain=c(rep("A",1000),rep("B",1000),rep("C",1000))
toplot$timepoint=c(1:1000,1:1000,1:1000)

toplot=gather(toplot,"parameter","value",1:7)

p_worm=ggplot(toplot)+
  geom_line(aes(x=timepoint,y=value,col=chain))+
  facet_grid(parameter~.,scales="free")+
  theme_bw()+
  ggtitle("Mesobiotus - Negative Binomial")
p_dens=ggplot(toplot)+
  geom_density(aes(x=value,col=chain))+
  facet_wrap(parameter~.,scales="free")+
  theme_bw()+ggtitle("Mesobiotus - Negative Binomial")
p_worm
```

```
p_dens
```

```
toplot=data.frame(do.call(rbind,as.mcmc(mod.pois.mes)))
toplot$chain=c(rep("A",1000),rep("B",1000),rep("C",1000))
toplot$timepoint=c(1:1000,1:1000,1:1000)

toplot=gather(toplot,"parameter","value",1:6)

p_worm=ggplot(toplot)+
  geom_line(aes(x=timepoint,y=value,col=chain))+
  facet_grid(parameter~.,scales="free")+
  theme_bw()+
  ggtitle("Mesobiotus - Poisson")
p_dens=ggplot(toplot)+
  geom_density(aes(x=value,col=chain))+
  facet_wrap(parameter~.,scales="free")+
  theme_bw()+ggtitle("Mesobiotus - Poisson")
p_worm
```

```
p_dens
```

```
toplot=data.frame(do.call(rbind,as.mcmc(mod.nb.dip)))
toplot$chain=c(rep("A",1000),rep("B",1000),rep("C",1000))
toplot$timepoint=c(1:1000,1:1000,1:1000)

toplot=gather(toplot,"parameter","value",1:7)

p_worm=ggplot(toplot)+
  geom_line(aes(x=timepoint,y=value,col=chain))+
  facet_grid(parameter~.,scales="free")+
  theme_bw()+
  ggtitle("Diphascon - Negative Binomial")
p_dens=ggplot(toplot)+
  geom_density(aes(x=value,col=chain))+
  facet_wrap(parameter~.,scales="free")+
  theme_bw()+ggtitle("Diphascon - Negative Binomial")
p_worm
```

```
p_dens
```

```
toplot=data.frame(do.call(rbind,as.mcmc(mod.pois.dip)))
toplot$chain=c(rep("A",1000),rep("B",1000),rep("C",1000))
toplot$timepoint=c(1:1000,1:1000,1:1000)

toplot=gather(toplot,"parameter","value",1:6)

p_worm=ggplot(toplot)+
  geom_line(aes(x=timepoint,y=value,col=chain))+
  facet_grid(parameter~.,scales="free")+
  theme_bw()+
  ggtitle("Diphascon - Poisson")
p_dens=ggplot(toplot)+
  geom_density(aes(x=value,col=chain))+
  facet_wrap(parameter~.,scales="free")+
  theme_bw()+ggtitle("Diphascon - Poisson")
p_worm
```

```
p_dens
```

NB: in the Negative Binomial on number of Taxa the r parameter is really large and almost take the shape of the prior distribution. I tried to increase the prior from dunif(0,100) to dunif(0,1000), however the posterior distribution span all of it anyway. This is not really a problem as with high r values, the Negative Binomial distribution approximates a Poisson distribution that was found to have a lower DIC.

##Appendix 2: plots of temperature and soil moisture in the warming chambers during study period

```
environmental_measures=read.csv("hf113-02-hf-chamber-since-2009.csv",header=T)
environmental_measures$cat.avg = rowMeans(environmental_measures[,11:12], na.rm=T)

environmental_measures_b = environmental_measures[,c(2,4,10,20,41)]

environmental_measures_aggregated = aggregate(environmental_measures_b[,4:5],
                                              by=list(environmental_measures_b$year,environmental_measures_b$doy,environmental_measures_b$chamber),
                                              FUN=mean)
colnames(environmental_measures_aggregated) = c("year","doy","chamber","moisture","temperature")


ptemp_1 = ggplot(subset(environmental_measures_aggregated,year!=2009))+
  theme_bw()+
  geom_line(aes(x=doy,y=temperature,group=as.factor(chamber),color=as.factor(chamber)))+
  facet_grid(year~.)+
  ggtitle("Air temperature")+
  xlab("Day of the year")+
  ylab("Temperature C")+
  theme(legend.position = "none")

pmoist_1 = ggplot(subset(environmental_measures_aggregated,year!=2009))+
  theme_bw()+
  geom_line(aes(x=doy,y=moisture,group=as.factor(chamber),color=as.factor(chamber)))+
  facet_grid(year~.)+
  ggtitle("Soil moisture")+
  xlab("Day of the year")+
  ylab("Moisture v/v")

ptemp_1|pmoist_1
```

```
environmental_measures_byday_min = aggregate(environmental_measures_aggregated[,4:5], 
                                         by = list(environmental_measures_aggregated$year, environmental_measures_aggregated$doy),
                                         FUN = min)

colnames(environmental_measures_byday_min) = c("year","doy","mean_moisture","mean_temperature")
environmental_measures_merged = merge(environmental_measures_aggregated,environmental_measures_byday_min)

environmental_measures_byday_sd = aggregate(environmental_measures_aggregated[,4:5], 
                                             by = list(environmental_measures_aggregated$year, environmental_measures_aggregated$doy),
                                             FUN = sd)


colnames(environmental_measures_byday_sd) = c("year","doy","sd_moisture","sd_temperature")
environmental_measures_merged = merge(environmental_measures_merged,environmental_measures_byday_sd)

environmental_measures_merged$temp_centered = (environmental_measures_merged$temperature - environmental_measures_merged$mean_temperature)/environmental_measures_merged$sd_temperature
environmental_measures_merged$moist_centered = (environmental_measures_merged$moisture - environmental_measures_merged$mean_moisture)/environmental_measures_merged$sd_moisture


environmental_measures_merged = subset(environmental_measures_merged, year != 2009)

ptemp_centered = ggplot(environmental_measures_merged)+
  theme_bw()+
  geom_line(aes(x=doy,y=temp_centered,group=as.factor(chamber),color=as.factor(chamber)))+
  facet_grid(year~.)

pmoist_centered = ggplot(environmental_measures_merged)+
  theme_bw()+
  geom_line(aes(x=doy,y=moist_centered,group=as.factor(chamber),color=as.factor(chamber)))+
  facet_grid(year~.)

ptemp_centered|pmoist_centered
```

##Appendix 3: ptable with number of individuals by taxa found in each chamber and temperature and soil moisture data
