# Supplementary material for: The toughest animals of the Earth versus global warming: Effects of long‐term experimental warming on tardigrade community structure of a temperate deciduous forest
Source: Ecol Evol. 2021 Jun 29;11(14):9856–63. doi: 10.1002/ece3.7816 (PMC8293726; doi:10.1002/ece3.7816)
Supplement: Supplementary file 1 — Appendix S1‐S2 [file ECE3-11-9856-s002.zip › SM.02.html]

Tardigrades in warming chambers - Soil temperature


# Tardigrades in warming chambers - Soil temperature

#### 2020-10-01

Data analysis of the “Tardigrades in Duke forest warming chambers project” for the publication: Vecchi M., Kossi Adakpo L., Dunn R.R., Nichols L.M., Penick C.A., Sanders N.J., Rebecchi L., Guidetti R. (2021). The toughest animals of the Earth versus global warming: Effects of long‐term experimental warming on tardigrade community structure of a temperate deciduous forest. *Ecology And Evolution*. Supplementary file S2.

Load all the libraries I need and get the info on the session with all the packages versions

```
library(R2jags) #running the models
library(tidyverse)
library(tidyr)  #sorting the models outputs for plotting
library(ggplot2) #plotting
library(ggridges) #plotting
library(DT) #show interactive table in the html file
library(vegan)
library(bayestestR)
library(robustbase)
library(patchwork)
sessionInfo()
```

```
## R version 4.0.2 (2020-06-22)
## Platform: x86_64-apple-darwin17.0 (64-bit)
## Running under: macOS  10.16
## 
## Matrix products: default
## BLAS:   /Library/Frameworks/R.framework/Versions/4.0/Resources/lib/libRblas.dylib
## LAPACK: /Library/Frameworks/R.framework/Versions/4.0/Resources/lib/libRlapack.dylib
## 
## locale:
## [1] en_US.UTF-8/en_US.UTF-8/en_US.UTF-8/C/en_US.UTF-8/en_US.UTF-8
## 
## attached base packages:
## [1] stats     graphics  grDevices utils     datasets  methods   base     
## 
## other attached packages:
##  [1] patchwork_1.1.0   robustbase_0.93-7 bayestestR_0.8.2  vegan_2.5-6      
##  [5] lattice_0.20-41   permute_0.9-5     DT_0.17           ggridges_0.5.2   
##  [9] forcats_0.5.0     stringr_1.4.0     dplyr_1.0.0       purrr_0.3.4      
## [13] readr_1.3.1       tidyr_1.1.0       tibble_3.0.1      ggplot2_3.3.2    
## [17] tidyverse_1.3.0   R2jags_0.6-1      rjags_4-10        coda_0.19-3      
## 
## loaded via a namespace (and not attached):
##  [1] httr_1.4.2        jsonlite_1.7.2    splines_4.0.2     modelr_0.1.8     
##  [5] assertthat_0.2.1  blob_1.2.1        cellranger_1.1.0  yaml_2.2.1       
##  [9] R2WinBUGS_2.1-21  pillar_1.4.4      backports_1.1.8   glue_1.4.1       
## [13] digest_0.6.25     rvest_0.3.5       colorspace_1.4-1  htmltools_0.5.1.1
## [17] Matrix_1.2-18     plyr_1.8.6        pkgconfig_2.0.3   broom_0.5.6      
## [21] haven_2.3.1       scales_1.1.1      mgcv_1.8-31       generics_0.1.0   
## [25] ellipsis_0.3.1    withr_2.4.1       cli_2.3.1         magrittr_1.5     
## [29] crayon_1.3.4      readxl_1.3.1      evaluate_0.14     fs_1.4.2         
## [33] nlme_3.1-148      MASS_7.3-51.6     xml2_1.3.2        tools_4.0.2      
## [37] hms_0.5.3         lifecycle_0.2.0   munsell_0.5.0     reprex_0.3.0     
## [41] cluster_2.1.0     compiler_4.0.2    rlang_0.4.10      grid_4.0.2       
## [45] rstudioapi_0.11   htmlwidgets_1.5.1 rmarkdown_2.3     boot_1.3-25      
## [49] gtable_0.3.0      abind_1.4-5       DBI_1.1.0         R6_2.4.1         
## [53] lubridate_1.7.9   knitr_1.29        insight_0.12.0    stringi_1.4.6    
## [57] parallel_4.0.2    Rcpp_1.0.4.6      vctrs_0.3.1       DEoptimR_1.0-8   
## [61] dbplyr_1.4.4      tidyselect_1.1.0  xfun_0.15
```

##Environmental measurements To get the chambers mean temperature and soil moisture download the raw measurements file hf113-02 from: http://harvardforest.fas.harvard.edu:8080/exist/apps/datasets/showData.html?id=hf113

###Load the environmental measures data

```
environmental_measures=read.csv("hf113-02-hf-chamber-since-2009.csv",header=T)
```

###Check the structure of the data

```
ncol(environmental_measures)
```

```
## [1] 40
```

```
nrow(environmental_measures)
```

```
## [1] 678786
```

```
colnames(environmental_measures)
```

```
##  [1] "datetime"  "year"      "decdate"   "doy"       "month"     "week"     
##  [7] "dom"       "hour"      "date"      "chamber"   "cat1.avg"  "cat2.avg" 
## [13] "catd.avg"  "csto1.avg" "csti1.avg" "csto2.avg" "csti2.avg" "cq.avg"   
## [19] "crh.avg"   "csm.avg"   "cat1.min"  "cat2.min"  "catd.min"  "csto1.min"
## [25] "csti1.min" "csto2.min" "csti2.min" "cq.min"    "crh.min"   "csm.min"  
## [31] "cat1.max"  "cat2.max"  "catd.max"  "csto1.max" "csti1.max" "csto2.max"
## [37] "csti2.max" "cq.max"    "crh.max"   "csm.max"
```

To be sure that there is nothing strange going on I plot the three column I´m interested in (cat1.avg, cat2.avg, csm.avg).

```
par(mfrow = c(1, 3))
boxplot(environmental_measures$csto1.avg, main = "Boxplot of csto1.avg", ylab = "Temperature Celsius")
boxplot(environmental_measures$csto2.avg, main = "Boxplot of csto2.avg", ylab = "Temperature Celsius")
boxplot(environmental_measures$csm.avg, main = "Boxplot of csm.avg", ylab = "Soil moisture v/v")
```

csto2 has a clear outlier (-80C) that probably comes from a instrument error so we reove that line of data.

```
environmental_measures = environmental_measures[environmental_measures$csto2.avg > -40,]
```

Check the boxplots again

```
par(mfrow = c(1, 3))
boxplot(environmental_measures$csto1.avg, main = "Boxplot of csto1.avg", ylab = "Temperature Celsius")
boxplot(environmental_measures$csto2.avg, main = "Boxplot of csto2.avg", ylab = "Temperature Celsius")
boxplot(environmental_measures$csm.avg, main = "Boxplot of csm.avg", ylab = "Soil moisture v/v")
```

The data looks OK, so I can select only the variables of my interest

###Get the average measure for each chamber

As there are two temperature measurements for each chamber.

```
environmental_measures$csto.avg = rowMeans(environmental_measures[,c(14,16)], na.rm=T)
```

Now I keep only the columns I am interested in (Chamber, cat.avg, csm.avg).

```
environmental_measures = environmental_measures[,c(10,20,41)]
colnames(environmental_measures)
```

```
## [1] "chamber"  "csm.avg"  "csto.avg"
```

Now I can average the measures by chamber

```
csto_mean_by_chamber = aggregate(environmental_measures$csto.avg, by = list(environmental_measures$chamber), FUN=mean, na.rm=T)
csm_mean_by_chamber = aggregate(environmental_measures$csm.avg, by = list(environmental_measures$chamber), FUN=mean, na.rm=T)
environmental_means = data.frame(chamber=1:12, csto.avg=csto_mean_by_chamber$x, csm.avg=csm_mean_by_chamber$x)
environmental_means
```

```
##    chamber  csto.avg   csm.avg
## 1        1 10.107233 0.1270569
## 2        2 10.149725 0.1291252
## 3        3  9.055800 0.1132096
## 4        4  8.547611 0.1088136
## 5        5 10.829556 0.1195994
## 6        6  8.528773 0.1484780
## 7        7  9.838477 0.1144209
## 8        8  9.317374 0.1157980
## 9        9  9.199582 0.1890335
## 10      10 10.343527 0.1783935
## 11      11  8.822807 0.1444840
## 12      12 10.025565 0.1915623
```

##Tardigrades dataset Now load the table with the number of individual recorded for each species in each chamber/replicate.

```
tardigrades_table=read.csv("tardigrades_warming_chambers_individuals.csv",header=T)
```

Let´s check the table:

```
head(tardigrades_table)
```

```
##   Chamber Mesobiotus Paramacrobiotus Macrobiotus Adropion Diphascon Hypsibius
## 1       1          1               0           0        1        11         0
## 2       1         16               0           0        2        24         0
## 3       2          9               0           0       66        82         0
## 4       2         13               0           0       54        15         0
## 5       3          4               0           0        0        33         0
## 6       3         21               0           5       12        12         1
##   Minibiotus Isohypsibius Milnesium Echiniscus Pilatobius Itaquascon
## 1          0            0         0          0          0          1
## 2          0            0         0          0          4          5
## 3          2            2         0          1          0          0
## 4          0            0         0          0          0          0
## 5          0            1         0          0          0          0
## 6          0            0         0          0          0          0
##   Pseudechiniscus
## 1               0
## 2               0
## 3               0
## 4               0
## 5               0
## 6               0
```

###Calculate the diversity index of tardigrade community Number of individuals

```
Individuals = rowSums(tardigrades_table[,2:ncol(tardigrades_table)])
```

Number of taxa

```
Taxa = rowSums(tardigrades_table[,2:ncol(tardigrades_table)]>0)
```

Shannon index

```
Shannon = vegan::diversity(tardigrades_table[,2:ncol(tardigrades_table)], index = "shannon")
```

##Let´s put all the data together I put all the environmental measures and community idexes in one table

First I duplicate each row of the environmental\_means table to accomodate for the two replicates for each chamber in the tardigrades table

```
environmental_means = environmental_means[rep(row.names(environmental_means) , 2) , ]
environmental_means = environmental_means[order(environmental_means$chamber),]
```

Then I attach all the data together

```
tardigrades_alldata=cbind(environmental_means,tardigrades_table,Individuals,Taxa,Shannon)
```

Here´s the complete data table

```
DT::datatable(tardigrades_alldata, rownames = FALSE)
```

## Variance partition

Estimate and plot variance partition

```
tardigrades_alldata$replicate=paste0("r",1:24)
anovas=summary(aov(data=tardigrades_alldata,cbind(Individuals,Taxa,Shannon,Mesobiotus,Adropion,Diphascon)~Chamber/replicate))
variances=lapply(anovas,function(x){x$`Sum Sq`})
variances.m=data.frame(matrix(unlist(variances)))
variances.m$level=rownames(anovas[[1]])
variances.m$var=c("Ind","Ind","Tax","Tax","Sha","Sha","Mesobiotus","Mesobiotus","Adropion","Adropion","Diphascon","Diphascon")
colnames(variances.m)[1]="value"
p=ggplot(variances.m)+geom_bar(aes(x=var,y=value,fill=level),position="fill",stat="identity")
p
```

## Bayesian GLMMs

###Individuals

Fort the individuals count i´ll try two different error families: negative binomial and poisson. The link function is log Negative Binomial and Poisson are chosen because they´re discrete probability distributions that goes from 0 to +Inf, like the indidividuals count index.

Let´s create the jags data for both the models:

```
data_jags=list(Nchamber=12,
                chamber=as.factor(tardigrades_alldata$Chamber),
                individuals=tardigrades_alldata$Individuals,
                temperature=as.numeric(scale(tardigrades_alldata$csto.avg)),
                moisture=as.numeric(scale(tardigrades_alldata$csm.avg)))
```

And run them

```
################################################
###### Individuals with Negative Binomial ######
################################################

mod_jags_nb <- function(){
# Priors
    alpha ~ dunif(-1000,1000)# intercept
    beta_temp ~ dunif(-1000,1000)# beta for temperature
    beta_moist ~ dunif(-1000,1000)# beta for soil moisture
    beta_inter ~ dunif(-1000,1000)# beta for interaction
    sigma_a ~ dunif(0, 100) # standard deviation of random effect (variance between sites)
    tau_a <- 1 / (sigma_a * sigma_a) # convert to precision
    r ~ dunif(0,100) # prior for overdispersion parameter  

# Random intercept for each chamber (random effect)
        for (j in 1:Nchamber){
        a[j] ~ dnorm(0, tau_a) 
    }

# Likelihood:
    for (i in 1:length(individuals)){
        mu[i] <- exp(alpha + a[chamber[i]] + beta_temp * temperature[i] + beta_moist * moisture[i] + beta_inter * temperature[i] * moisture[i]) #predicted values with log link
        p[i] <- r / ( r + mu[i]) #reparametrization of negbin parameters according to: http://doingbayesiandataanalysis.blogspot.com/2012/04/negative-binomial-reparameterization.html
        individuals[i] ~ dnegbin( p[i] , r )
    }
}

mod_params_nb <- c("alpha", "beta_temp", "beta_moist", "beta_inter", "sigma_a","r")


mod.nb.ind=jags(data=data_jags,parameters.to.save=mod_params_nb, model.file=mod_jags_nb,n.iter=10000000)

######################################
###### Individuals with Poisson ######
######################################

mod_jags_pois <- function(){
# Priors
    alpha ~ dunif(-1000,1000)# intercept
    beta_temp ~ dunif(-1000,1000)# beta for temperature
    beta_moist ~ dunif(-1000,1000)# beta for soil moisture
    beta_inter ~ dunif(-1000,1000)# beta for interaction
    sigma_a ~ dunif(0, 100) # standard deviation of random effect (variance between sites)
    tau_a <- 1 / (sigma_a * sigma_a) # convert to precision

# Random intercept for each chamber (random effect)
        for (j in 1:Nchamber){
        a[j] ~ dnorm(0, tau_a) 
    }

# Likelihood:
    for (i in 1:length(individuals)){
        mu[i] <- exp(alpha + a[chamber[i]] + beta_temp * temperature[i] + beta_moist * moisture[i] + beta_inter * temperature[i] * moisture[i]) #predicted values with log link
        individuals[i] ~ dpois(mu[i])
    }
}

mod_params_pois <- c("alpha", "beta_temp", "beta_moist", "beta_inter", "sigma_a")


mod.pois.ind=jags(data=data_jags,parameters.to.save=mod_params_pois, model.file=mod_jags_pois,n.iter=10000000)
```

Now check if the models converged and their DICS

```
mod.nb.ind
```

```
## Inference for Bugs model at "C:/Users/mavecchi/AppData/Local/Temp/RtmpSofaxe/model3854475a1a4f.txt", fit using jags,
##  3 chains, each with 1e+07 iterations (first 5e+06 discarded), n.thin = 5000
##  n.sims = 3000 iterations saved
##            mu.vect sd.vect    2.5%     25%     50%     75%   97.5%  Rhat n.eff
## alpha        4.197   0.222   3.765   4.053   4.191   4.333   4.649 1.002  1500
## beta_inter   0.517   0.287  -0.045   0.337   0.518   0.703   1.086 1.001  3000
## beta_moist   0.026   0.230  -0.434  -0.119   0.027   0.173   0.476 1.001  3000
## beta_temp    0.241   0.245  -0.252   0.085   0.245   0.399   0.719 1.001  3000
## r            1.491   0.429   0.802   1.190   1.440   1.739   2.415 1.001  3000
## sigma_a      0.337   0.267   0.015   0.132   0.287   0.469   1.003 1.001  3000
## deviance   252.291   3.915 245.873 249.615 251.797 254.450 261.383 1.001  3000
## 
## For each parameter, n.eff is a crude measure of effective sample size,
## and Rhat is the potential scale reduction factor (at convergence, Rhat=1).
## 
## DIC info (using the rule, pD = var(deviance)/2)
## pD = 7.7 and DIC = 260.0
## DIC is an estimate of expected predictive error (lower deviance is better).
```

```
autocorr.diag(as.mcmc(mod.nb.ind))
```

```
##                   alpha   beta_inter   beta_moist    beta_temp    deviance
## Lag 0       1.000000000  1.000000000  1.000000000  1.000000000  1.00000000
## Lag 5000   -0.009992133 -0.016747346  0.007349923  0.032018271 -0.01559086
## Lag 25000  -0.015797376 -0.010423799 -0.017471893 -0.020294459 -0.01400390
## Lag 50000  -0.001552669 -0.019568108 -0.018910868  0.010659829  0.05075637
## Lag 250000 -0.016693074 -0.003881317  0.016051080 -0.004196078 -0.03320892
##                       r     sigma_a
## Lag 0       1.000000000  1.00000000
## Lag 5000    0.006797271 -0.02270593
## Lag 25000  -0.035642367 -0.02274102
## Lag 50000   0.001667555  0.01029478
## Lag 250000  0.019871093 -0.02595563
```

```
mod.pois.ind
```

```
## Inference for Bugs model at "C:/Users/mavecchi/AppData/Local/Temp/RtmpSofaxe/model3854c7b1de4.txt", fit using jags,
##  3 chains, each with 1e+07 iterations (first 5e+06 discarded), n.thin = 5000
##  n.sims = 3000 iterations saved
##            mu.vect sd.vect    2.5%     25%     50%     75%   97.5%  Rhat n.eff
## alpha        4.055   0.192   3.671   3.938   4.057   4.169   4.434 1.002  1700
## beta_inter   0.487   0.243  -0.008   0.346   0.490   0.638   0.956 1.001  3000
## beta_moist   0.015   0.202  -0.382  -0.109   0.018   0.136   0.420 1.001  3000
## beta_temp    0.210   0.206  -0.187   0.088   0.212   0.329   0.627 1.002  1800
## sigma_a      0.631   0.219   0.364   0.483   0.585   0.722   1.198 1.003   920
## deviance   981.961   5.051 974.373 978.282 981.158 984.896 994.027 1.001  3000
## 
## For each parameter, n.eff is a crude measure of effective sample size,
## and Rhat is the potential scale reduction factor (at convergence, Rhat=1).
## 
## DIC info (using the rule, pD = var(deviance)/2)
## pD = 12.8 and DIC = 994.7
## DIC is an estimate of expected predictive error (lower deviance is better).
```

```
autocorr.diag(as.mcmc(mod.pois.ind))
```

```
##                   alpha   beta_inter   beta_moist    beta_temp     deviance
## Lag 0       1.000000000  1.000000000  1.000000000  1.000000000  1.000000000
## Lag 5000   -0.021536760  0.002590538 -0.007959644 -0.000594842  0.020878043
## Lag 25000   0.024367029  0.003110713  0.010545542 -0.003273922  0.013527345
## Lag 50000  -0.002683786  0.019959115 -0.012218718  0.027395362 -0.004114745
## Lag 250000  0.023771960 -0.008801700 -0.041302957 -0.017685407 -0.008238352
##                 sigma_a
## Lag 0       1.000000000
## Lag 5000    0.005919807
## Lag 25000  -0.033621051
## Lag 50000   0.014403918
## Lag 250000 -0.007457928
```

Despite the n.eff values are not ideal, the Rhat and autocorrelations looks fine, so I´ll keep those models.  
As the Negative Binomial model has a lower DIC the Poisson model will be discarded.

###Taxa

For the number of species I´ll try as well Negative Binomial and Poisson with a log link. Negative Binomial and Poisson are chosen because they´re discrete probability distributions that goes from 0 to +Inf, like the Taxa count index.

Let´s create the jags data for both the models:

```
data_jags=list(Nchamber=12,
                chamber=as.factor(tardigrades_alldata$Chamber),
                taxa=tardigrades_alldata$Taxa,
                temperature=as.numeric(scale(tardigrades_alldata$csto.avg)),
                moisture=as.numeric(scale(tardigrades_alldata$csm.avg)))
```

And run them

```
################################################
###### Taxa with Negative Binomial ######
################################################

mod_jags_nb <- function(){
  # Priors
  alpha ~ dunif(-1000,1000)# intercept
  beta_temp ~ dunif(-1000,1000)# beta for temperature
  beta_moist ~ dunif(-1000,1000)# beta for soil moisture
  beta_inter ~ dunif(-1000,1000)# beta for interaction
  sigma_a ~ dunif(0, 100) # standard deviation of random effect (variance between sites)
  tau_a <- 1 / (sigma_a * sigma_a) # convert to precision
  #r ~ dgamma(0.1 , 0.1) # prior for overdispersion parameter according to: http://doingbayesiandataanalysis.blogspot.com/2012/04/negative-binomial-reparameterization.html
  r ~ dunif(0,100) # prior for overdispersion parameter 
  
  # Random intercept for each chamber (random effect)
  for (j in 1:Nchamber){
    a[j] ~ dnorm(0, tau_a) 
  }
  
  # Likelihood:
  for (i in 1:length(taxa)){
    mu[i] <- exp(alpha + a[chamber[i]] + beta_temp * temperature[i] + beta_moist * moisture[i] + beta_inter * temperature[i] * moisture[i]) #predicted values with log link
    p[i] <- r / ( r + mu[i]) #reparametrization of negbin parameters according to: http://doingbayesiandataanalysis.blogspot.com/2012/04/negative-binomial-reparameterization.html
    taxa[i] ~ dnegbin( p[i] , r )
  }
}

mod_params_nb <- c("alpha", "beta_temp", "beta_moist", "beta_inter", "sigma_a","r")

mod.nb.taxa=jags(data=data_jags,parameters.to.save=mod_params_nb, model.file=mod_jags_nb,n.iter=10000000)

######################################
###### Taxa with Poisson ######
######################################

mod_jags_pois <- function(){
  # Priors
  alpha ~ dunif(-1000,1000)# intercept
  beta_temp ~ dunif(-1000,1000)# beta for temperature
  beta_moist ~ dunif(-1000,1000)# beta for soil moisture
  beta_inter ~ dunif(-1000,1000)# beta for interaction
  sigma_a ~ dunif(0, 100) # standard deviation of random effect (variance between sites)
  tau_a <- 1 / (sigma_a * sigma_a) # convert to precision
  
  # Random intercept for each chamber (random effect)
  for (j in 1:Nchamber){
    a[j] ~ dnorm(0, tau_a) 
  }
  
  # Likelihood:
  for (i in 1:length(taxa)){
    mu[i] <- exp(alpha + a[chamber[i]] + beta_temp * temperature[i] + beta_moist * moisture[i] + beta_inter * temperature[i] * moisture[i]) #predicted values with log link
    taxa[i] ~ dpois(mu[i])
  }
}

mod_params_pois <- c("alpha", "beta_temp", "beta_moist", "beta_inter", "sigma_a")


mod.pois.taxa=jags(data=data_jags,parameters.to.save=mod_params_pois, model.file=mod_jags_pois,n.iter=10000000)
```

Now check if the models converged and their DICS

```
mod.nb.taxa
```

```
## Inference for Bugs model at "C:/Users/mavecchi/AppData/Local/Temp/RtmpSofaxe/model3854219e887.txt", fit using jags,
##  3 chains, each with 1e+07 iterations (first 5e+06 discarded), n.thin = 5000
##  n.sims = 3000 iterations saved
##            mu.vect sd.vect   2.5%    25%    50%    75%   97.5%  Rhat n.eff
## alpha        1.433   0.115  1.204  1.359  1.434  1.508   1.655 1.001  2200
## beta_inter  -0.029   0.147 -0.320 -0.123 -0.029  0.066   0.263 1.001  3000
## beta_moist   0.087   0.117 -0.140  0.011  0.088  0.163   0.314 1.001  3000
## beta_temp    0.010   0.122 -0.241 -0.067  0.014  0.089   0.246 1.001  3000
## r           60.089  25.013 13.619 39.868 61.778 81.653  98.308 1.003   830
## sigma_a      0.139   0.117  0.005  0.054  0.111  0.195   0.428 1.001  3000
## deviance    96.230   3.177 91.919 93.890 95.638 97.980 104.093 1.001  3000
## 
## For each parameter, n.eff is a crude measure of effective sample size,
## and Rhat is the potential scale reduction factor (at convergence, Rhat=1).
## 
## DIC info (using the rule, pD = var(deviance)/2)
## pD = 5.0 and DIC = 101.3
## DIC is an estimate of expected predictive error (lower deviance is better).
```

```
autocorr.diag(as.mcmc(mod.nb.taxa))
```

```
##                   alpha   beta_inter   beta_moist   beta_temp     deviance
## Lag 0       1.000000000  1.000000000  1.000000000  1.00000000  1.000000000
## Lag 5000   -0.002539787  0.021974735  0.010829353  0.02831848 -0.008945329
## Lag 25000  -0.028444820 -0.009930821 -0.007808142 -0.01164473 -0.011227751
## Lag 50000   0.008810376 -0.019508518  0.022694550 -0.02049243 -0.001176737
## Lag 250000 -0.003686778  0.010972279  0.009098465 -0.02602446  0.014650046
##                       r     sigma_a
## Lag 0       1.000000000  1.00000000
## Lag 5000   -0.005150594  0.03219696
## Lag 25000   0.038702997  0.01904569
## Lag 50000   0.013353365 -0.01110573
## Lag 250000 -0.009066915 -0.00265442
```

```
mod.pois.taxa
```

```
## Inference for Bugs model at "C:/Users/mavecchi/AppData/Local/Temp/RtmpSofaxe/model38542f284096.txt", fit using jags,
##  3 chains, each with 1e+07 iterations (first 5e+06 discarded), n.thin = 5000
##  n.sims = 3000 iterations saved
##            mu.vect sd.vect   2.5%    25%    50%    75%   97.5%  Rhat n.eff
## alpha        1.424   0.111  1.199  1.353  1.426  1.499   1.634 1.001  3000
## beta_inter  -0.029   0.142 -0.311 -0.122 -0.027  0.063   0.246 1.001  3000
## beta_moist   0.089   0.114 -0.130  0.013  0.088  0.163   0.317 1.001  3000
## beta_temp    0.010   0.120 -0.232 -0.068  0.008  0.087   0.247 1.001  3000
## sigma_a      0.136   0.114  0.004  0.050  0.109  0.190   0.432 1.001  3000
## deviance    95.274   3.280 90.942 92.869 94.649 96.824 103.433 1.001  3000
## 
## For each parameter, n.eff is a crude measure of effective sample size,
## and Rhat is the potential scale reduction factor (at convergence, Rhat=1).
## 
## DIC info (using the rule, pD = var(deviance)/2)
## pD = 5.4 and DIC = 100.7
## DIC is an estimate of expected predictive error (lower deviance is better).
```

```
autocorr.diag(as.mcmc(mod.pois.taxa))
```

```
##                    alpha   beta_inter   beta_moist     beta_temp    deviance
## Lag 0       1.0000000000  1.000000000  1.000000000  1.0000000000 1.000000000
## Lag 5000    0.0039125531  0.001102618  0.007741939 -0.0172628450 0.003450317
## Lag 25000  -0.0003850094 -0.003898968  0.030819023 -0.0003487422 0.010886796
## Lag 50000   0.0018199362 -0.024966097 -0.013286364  0.0127526688 0.006237299
## Lag 250000 -0.0044739085  0.003944660 -0.026196004 -0.0020748677 0.012007926
##                sigma_a
## Lag 0       1.00000000
## Lag 5000   -0.01170654
## Lag 25000  -0.00148852
## Lag 50000  -0.01999384
## Lag 250000  0.03267988
```

Despite the n.eff values are not ideal, the Rhat and autocorrelations looks fine, so I´ll keep those models.  
As the Poisson model has a lower DIC the Negative Binomial model will be discarded.

###Shannon

For the Shannon index I´ll use a Gamma family with a log link. Gamma is chosen because it´s a continuous probability distributions that goes from 0 to +Inf, like the Shannon index.

Let´s create the jags data:

```
data_jags=list(Nchamber=12,
                chamber=as.factor(tardigrades_alldata$Chamber),
            shannon=tardigrades_alldata$Shannon,
                temperature=as.numeric(scale(tardigrades_alldata$csto.avg)),
                moisture=as.numeric(scale(tardigrades_alldata$csm.avg)))
```

And run them

```
################################################
###### Shannon with Gamma ######
################################################

mod_jags <- function(){
  # Priors
  alpha ~ dunif(-1000,1000)# intercept
  beta_temp ~ dunif(-1000,1000)# beta for temperature
  beta_moist ~ dunif(-1000,1000)# beta for soil moisture
  beta_inter ~ dunif(-1000,1000)# beta for interaction
  sigma_a ~ dunif(0, 100) # standard deviation of random effect (variance between sites)
  tau_a <- 1 / (sigma_a * sigma_a) # convert to precision
  sigma ~ dunif(0, 100) # standard deviation of Gamma distribution
  
  # Random intercept for each chamber (random effect)
  for (j in 1:Nchamber){
    a[j] ~ dnorm(0, tau_a) 
  }
  
  # Likelihood:
  for (i in 1:length(shannon)){
    mu[i] <- exp(alpha + a[chamber[i]] + beta_temp * temperature[i] + beta_moist * moisture[i] + beta_inter * temperature[i] * moisture[i]) #predicted values with log link
    sh[i] <- pow(mu[i],2)/pow(sigma,2) # Shape parameter of Gamma distribution reparametrized according to : http://doingbayesiandataanalysis.blogspot.com/2012/08/gamma-likelihood-parameterized-by-mode.html
    ra[i] <- mu[i]/pow(sigma,2)        # Rate parameter of Gamma distribution reparametrized according to : http://doingbayesiandataanalysis.blogspot.com/2012/08/gamma-likelihood-parameterized-by-mode.html
    shannon[i] ~ dgamma(sh[i],ra[i])
  }
}

mod_params <- c("alpha", "beta_temp", "beta_moist", "beta_inter", "sigma_a","sigma")


mod.sha=jags(data=data_jags,parameters.to.save=mod_params, model.file=mod_jags,n.iter=10000000)
```

Now check if the model converged

```
mod.sha
```

```
## Inference for Bugs model at "C:/Users/mavecchi/AppData/Local/Temp/RtmpSofaxe/model385467322276.txt", fit using jags,
##  3 chains, each with 1e+07 iterations (first 5e+06 discarded), n.thin = 5000
##  n.sims = 3000 iterations saved
##            mu.vect sd.vect   2.5%    25%    50%    75%  97.5%  Rhat n.eff
## alpha       -0.122   0.089 -0.305 -0.176 -0.120 -0.066  0.054 1.001  3000
## beta_inter  -0.120   0.105 -0.340 -0.187 -0.117 -0.054  0.076 1.001  3000
## beta_moist  -0.011   0.085 -0.186 -0.064 -0.008  0.044  0.152 1.001  3000
## beta_temp    0.008   0.084 -0.161 -0.046  0.008  0.059  0.179 1.001  3000
## sigma        0.333   0.060  0.239  0.290  0.324  0.365  0.475 1.001  3000
## sigma_a      0.104   0.088  0.004  0.040  0.083  0.142  0.321 1.004  2100
## deviance    13.073   4.021  7.377 10.047 12.296 15.378 22.774 1.001  3000
## 
## For each parameter, n.eff is a crude measure of effective sample size,
## and Rhat is the potential scale reduction factor (at convergence, Rhat=1).
## 
## DIC info (using the rule, pD = var(deviance)/2)
## pD = 8.1 and DIC = 21.2
## DIC is an estimate of expected predictive error (lower deviance is better).
```

```
autocorr.diag(as.mcmc(mod.sha))
```

```
##                    alpha   beta_inter   beta_moist    beta_temp    deviance
## Lag 0       1.0000000000  1.000000000  1.000000000  1.000000000  1.00000000
## Lag 5000   -0.0143204683 -0.003836507 -0.008705457 -0.012842748 -0.02267213
## Lag 25000  -0.0013380324  0.008120296  0.032031253  0.004848860  0.01990570
## Lag 50000   0.0009745143  0.007318882 -0.043571620  0.004736220 -0.01451624
## Lag 250000  0.0084134757 -0.006091118 -0.032690583  0.006226691  0.03241414
##                  sigma       sigma_a
## Lag 0       1.00000000  1.0000000000
## Lag 5000   -0.01585340 -0.0141251421
## Lag 25000  -0.03021967 -0.0001736137
## Lag 50000   0.03837065 -0.0106435253
## Lag 250000  0.02912621 -0.0041463441
```

### Adropion

For the Adropion individuals i´ll try two different error families: negative binomial and poisson. For both of them I´ll test with or without zero inflation as there are some replicates with no Adropion individuals. The link function is log

Let´s create the jags data:

```
data_jags=list(Nchamber=12,
               chamber=as.factor(tardigrades_alldata$Chamber),
               individuals=tardigrades_alldata$Adropion,
               temperature=as.numeric(scale(tardigrades_alldata$csto.avg)),
               moisture=as.numeric(scale(tardigrades_alldata$csm.avg)))
```

And run them:

```
################################################
###### Adropion with Negative Binomial     ######
################################################

mod_jags_nb <- function(){
  # Priors
  alpha ~ dunif(-1000,1000)# intercept
  beta_temp ~ dunif(-1000,1000)# beta for temperature
  beta_moist ~ dunif(-1000,1000)# beta for soil moisture
  beta_inter ~ dunif(-1000,1000)# beta for interaction
  sigma_a ~ dunif(0, 100) # standard deviation of random effect (variance between sites)
  tau_a <- 1 / (sigma_a * sigma_a) # convert to precision
  r ~ dgamma(0.1 , 0.1) # prior for overdispersion parameter according to: http://doingbayesiandataanalysis.blogspot.com/2012/04/negative-binomial-reparameterization.html
  
  # Random intercept for each chamber (random effect)
  for (j in 1:Nchamber){
    a[j] ~ dnorm(0, tau_a) 
  }
  
  # Likelihood:
  for (i in 1:length(individuals)){
    mu[i] <- exp(alpha + a[chamber[i]] + beta_temp * temperature[i] + beta_moist * moisture[i] + beta_inter * temperature[i] * moisture[i]) #predicted values with log link
    p[i] <- r / ( r + mu[i]) #reparametrization of negbin parameters according to: http://doingbayesiandataanalysis.blogspot.com/2012/04/negative-binomial-reparameterization.html
    individuals[i] ~ dnegbin( p[i] , r )
  }
}

mod_params_nb <- c("alpha", "beta_temp", "beta_moist", "beta_inter", "sigma_a","r")


mod.nb.adr=jags(data=data_jags,parameters.to.save=mod_params_nb, model.file=mod_jags_nb,n.iter=100000)

##################################
###### Adropion with Poisson ######
##################################

mod_jags_pois <- function(){
  # Priors
  alpha ~ dunif(-1000,1000)# intercept
  beta_temp ~ dunif(-1000,1000)# beta for temperature
  beta_moist ~ dunif(-1000,1000)# beta for soil moisture
  beta_inter ~ dunif(-1000,1000)# beta for interaction
  sigma_a ~ dunif(0, 100) # standard deviation of random effect (variance between sites)
  tau_a <- 1 / (sigma_a * sigma_a) # convert to precision
  
  # Random intercept for each chamber (random effect)
  for (j in 1:Nchamber){
    a[j] ~ dnorm(0, tau_a) 
  }
  
  # Likelihood:
  for (i in 1:length(individuals)){
    mu[i] <- exp(alpha + a[chamber[i]] + beta_temp * temperature[i] + beta_moist * moisture[i] + beta_inter * temperature[i] * moisture[i]) #predicted values with log link
    individuals[i] ~ dpois(mu[i])
  }
}

mod_params_pois <- c("alpha", "beta_temp", "beta_moist", "beta_inter", "sigma_a")


mod.pois.adr=jags(data=data_jags,parameters.to.save=mod_params_pois, model.file=mod_jags_pois,n.iter=100000)

####################################################################
###### Adropion with Negative Binomial with zero inflation     ######
####################################################################

mod_jags_nb <- function(){
  # Priors
  alpha ~ dunif(-1000,1000)# intercept
  beta_temp ~ dunif(-1000,1000)# beta for temperature
  beta_moist ~ dunif(-1000,1000)# beta for soil moisture
  beta_inter ~ dunif(-1000,1000)# beta for interaction
  sigma_a ~ dunif(0, 100) # standard deviation of random effect (variance between sites)
  tau_a <- 1 / (sigma_a * sigma_a) # convert to precision
  r ~ dgamma(0.1 , 0.1) # prior for overdispersion parameter according to: http://doingbayesiandataanalysis.blogspot.com/2012/04/negative-binomial-reparameterization.html
  psi ~ dunif(0, 1) # proportion of non-zeros
  psi_zeros <- 1-psi #proportion of zeros
  
  # Random intercept for each chamber (random effect)
  for (j in 1:Nchamber){
    a[j] ~ dnorm(0, tau_a) 
  }
  
  # Likelihood:
  for (i in 1:length(individuals)){
    mu[i] <- exp(alpha + a[chamber[i]] + beta_temp * temperature[i] + beta_moist * moisture[i] + beta_inter * temperature[i] * moisture[i]) #predicted values with log link
    
    z[i] ~ dbern(psi) #zero inflation
    mu_zeroinfl[i] <- mu[i]*z[i] + 0.00001*(1-z[i]) ## hack required for Rjags -- otherwise 'incompatible'-error
    
    p[i] <- r / ( r + mu_zeroinfl[i]) #reparametrization of negbin parameters according to: http://doingbayesiandataanalysis.blogspot.com/2012/04/negative-binomial-reparameterization.html
    individuals[i] ~ dnegbin( p[i] , r )
  }
}

mod_params_nb <- c("alpha", "beta_temp", "beta_moist", "beta_inter", "sigma_a","r","psi_zeros")

mod.nb.zero.adr=jags(data=data_jags,parameters.to.save=mod_params_nb, model.file=mod_jags_nb,n.iter=100000)

######################################################
###### Adropion with Poisson with zero inflation ######
######################################################

mod_jags_pois <- function(){
  # Priors
  alpha ~ dunif(-1000,1000)# intercept
  beta_temp ~ dunif(-1000,1000)# beta for temperature
  beta_moist ~ dunif(-1000,1000)# beta for soil moisture
  beta_inter ~ dunif(-1000,1000)# beta for interaction
  sigma_a ~ dunif(0, 100) # standard deviation of random effect (variance between sites)
  tau_a <- 1 / (sigma_a * sigma_a) # convert to precision
  psi ~ dunif(0, 1) # proportion of non-zeros
  psi_zeros <- 1-psi #proportion of zeros
  
  # Random intercept for each chamber (random effect)
  for (j in 1:Nchamber){
    a[j] ~ dnorm(0, tau_a) 
  }
  
  # Likelihood:
  for (i in 1:length(individuals)){
    mu[i] <- exp(alpha + a[chamber[i]] + beta_temp * temperature[i] + beta_moist * moisture[i] + beta_inter * temperature[i] * moisture[i]) #predicted values with log link
    
    z[i] ~ dbern(psi) #zero inflation
    mu_zeroinfl[i] <- mu[i]*z[i] + 0.00001*(1-z[i]) ## hack required for Rjags -- otherwise 'incompatible'-error
    
    individuals[i] ~ dpois(mu_zeroinfl[i])
  }
}

mod_params_pois <- c("alpha", "beta_temp", "beta_moist", "beta_inter", "sigma_a","psi_zeros")


mod.pois.zero.adr=jags(data=data_jags,parameters.to.save=mod_params_pois, model.file=mod_jags_pois,n.iter=100000)
```

Now check if the models converged

Negative Binomial

```
mod.nb.adr
```

```
## Inference for Bugs model at "C:/Users/mavecchi/AppData/Local/Temp/RtmpSofaxe/model385427dc69fa.txt", fit using jags,
##  3 chains, each with 1e+05 iterations (first 50000 discarded), n.thin = 50
##  n.sims = 3000 iterations saved
##            mu.vect sd.vect    2.5%     25%     50%     75%   97.5%  Rhat n.eff
## alpha        3.249   0.925   1.482   2.738   3.251   3.761   5.048 1.002  2600
## beta_inter  -0.360   1.265  -3.209  -1.037  -0.278   0.439   1.881 1.001  3000
## beta_moist   0.489   0.895  -1.119  -0.049   0.431   0.965   2.506 1.001  3000
## beta_temp    0.585   0.955  -1.265   0.058   0.579   1.102   2.463 1.002  2500
## r            0.281   0.108   0.126   0.205   0.264   0.337   0.540 1.001  3000
## sigma_a      1.658   1.257   0.067   0.781   1.449   2.230   4.715 1.001  3000
## deviance   177.836   6.125 166.045 173.705 177.928 181.733 190.480 1.001  3000
## 
## For each parameter, n.eff is a crude measure of effective sample size,
## and Rhat is the potential scale reduction factor (at convergence, Rhat=1).
## 
## DIC info (using the rule, pD = var(deviance)/2)
## pD = 18.8 and DIC = 196.6
## DIC is an estimate of expected predictive error (lower deviance is better).
```

```
autocorr.diag(as.mcmc(mod.nb.adr))
```

```
##                alpha  beta_inter   beta_moist   beta_temp    deviance
## Lag 0     1.00000000  1.00000000  1.000000000 1.000000000  1.00000000
## Lag 50    0.07003592  0.03153648  0.023473346 0.056986883  0.06638970
## Lag 250   0.00244025 -0.02265688  0.007755197 0.006181275 -0.01428676
## Lag 500  -0.03972584 -0.03711545 -0.021979389 0.027536726  0.00251131
## Lag 2500  0.01641312 -0.01512988  0.007502656 0.038907721  0.01138232
##                    r      sigma_a
## Lag 0     1.00000000  1.000000000
## Lag 50    0.02820163  0.276331493
## Lag 250  -0.02774042 -0.008965598
## Lag 500  -0.02238788 -0.008808219
## Lag 2500  0.01408782  0.010626559
```

Poisson

```
mod.pois.adr
```

```
## Inference for Bugs model at "C:/Users/mavecchi/AppData/Local/Temp/RtmpSofaxe/model385455122ff7.txt", fit using jags,
##  3 chains, each with 1e+05 iterations (first 50000 discarded), n.thin = 50
##  n.sims = 3000 iterations saved
##            mu.vect sd.vect    2.5%     25%     50%     75%   97.5%  Rhat n.eff
## alpha        2.218   0.867   0.447   1.763   2.250   2.693   3.872 1.060   120
## beta_inter  -0.710   1.227  -3.686  -1.310  -0.601   0.038   1.465 1.038   200
## beta_moist   0.383   0.954  -1.359  -0.183   0.313   0.907   2.474 1.103    26
## beta_temp    0.528   0.759  -0.885   0.040   0.505   0.980   2.145 1.009   290
## sigma_a      2.618   0.993   1.396   1.934   2.401   3.045   5.176 1.001  3000
## deviance   700.089   4.856 692.414 696.604 699.512 702.820 711.173 1.001  3000
## 
## For each parameter, n.eff is a crude measure of effective sample size,
## and Rhat is the potential scale reduction factor (at convergence, Rhat=1).
## 
## DIC info (using the rule, pD = var(deviance)/2)
## pD = 11.8 and DIC = 711.9
## DIC is an estimate of expected predictive error (lower deviance is better).
```

```
autocorr.diag(as.mcmc(mod.pois.adr))
```

```
##               alpha beta_inter beta_moist   beta_temp     deviance    sigma_a
## Lag 0    1.00000000  1.0000000  1.0000000  1.00000000  1.000000000 1.00000000
## Lag 50   0.92593248  0.9436782  0.9624655  0.86758960  0.032626044 0.45644184
## Lag 250  0.69934442  0.7538211  0.8284501  0.48851799 -0.013957611 0.41655674
## Lag 500  0.50030606  0.5458486  0.6779216  0.22792982  0.019999501 0.31738486
## Lag 2500 0.02807493  0.1205296  0.1859227 -0.07889021 -0.008702367 0.02496851
```

Zero-inflated negative binomial

```
mod.nb.zero.adr
```

```
## Inference for Bugs model at "C:/Users/mavecchi/AppData/Local/Temp/RtmpSofaxe/model38544d96656b.txt", fit using jags,
##  3 chains, each with 1e+05 iterations (first 50000 discarded), n.thin = 50
##  n.sims = 3000 iterations saved
##            mu.vect sd.vect    2.5%     25%     50%     75%   97.5%  Rhat n.eff
## alpha        3.303   0.824   1.639   2.820   3.299   3.770   4.985 1.002  3000
## beta_inter  -0.127   1.316  -2.995  -0.859  -0.040   0.749   2.109 1.001  3000
## beta_moist   0.370   0.905  -1.261  -0.166   0.323   0.835   2.477 1.002  3000
## beta_temp    0.487   0.896  -1.349  -0.046   0.476   1.002   2.280 1.001  3000
## psi_zeros    0.144   0.103   0.006   0.060   0.126   0.207   0.384 1.001  3000
## r            0.422   0.234   0.153   0.262   0.366   0.511   1.031 1.001  3000
## sigma_a      1.643   1.181   0.071   0.794   1.438   2.232   4.523 1.011   650
## deviance   168.374  10.258 146.725 161.352 169.356 175.878 186.056 1.001  3000
## 
## For each parameter, n.eff is a crude measure of effective sample size,
## and Rhat is the potential scale reduction factor (at convergence, Rhat=1).
## 
## DIC info (using the rule, pD = var(deviance)/2)
## pD = 52.6 and DIC = 221.0
## DIC is an estimate of expected predictive error (lower deviance is better).
```

```
autocorr.diag(as.mcmc(mod.nb.zero.adr))
```

```
##                alpha   beta_inter   beta_moist    beta_temp      deviance
## Lag 0     1.00000000  1.000000000  1.000000000  1.000000000  1.000000e+00
## Lag 50    0.03069492  0.061330687  0.031655851  0.001119805  3.314799e-02
## Lag 250   0.01527274 -0.002504143 -0.017699712 -0.007531526 -1.470943e-02
## Lag 500  -0.03444006 -0.009321729 -0.022780485 -0.023946575  6.278723e-05
## Lag 2500 -0.03209868 -0.019914349 -0.006518643 -0.012997296  8.088690e-03
##            psi_zeros             r      sigma_a
## Lag 0    1.000000000  1.0000000000  1.000000000
## Lag 50   0.006308056  0.0256437567  0.281482948
## Lag 250  0.018735499  0.0184687719  0.007602271
## Lag 500  0.008037842 -0.0024438855 -0.012872065
## Lag 2500 0.007307341  0.0004001103 -0.015091358
```

Zero-inflated poisson

```
mod.pois.zero.adr
```

```
## Inference for Bugs model at "C:/Users/mavecchi/AppData/Local/Temp/RtmpSofaxe/model38543d5440df.txt", fit using jags,
##  3 chains, each with 1e+05 iterations (first 50000 discarded), n.thin = 50
##  n.sims = 3000 iterations saved
##            mu.vect sd.vect    2.5%     25%     50%     75%   97.5%  Rhat n.eff
## alpha        2.822   0.946   0.658   2.394   2.861   3.300   4.736 1.039   250
## beta_inter  -0.103   1.181  -2.743  -0.757  -0.012   0.698   1.974 1.012  1200
## beta_moist   0.038   0.956  -1.753  -0.525  -0.060   0.499   2.334 1.048    67
## beta_temp    0.118   0.878  -1.644  -0.434   0.134   0.660   1.946 1.041    53
## psi_zeros    0.322   0.094   0.148   0.256   0.319   0.386   0.512 1.001  3000
## sigma_a      2.511   1.070   1.255   1.778   2.242   2.953   5.235 1.008   450
## deviance   413.713   5.045 406.068 409.991 413.107 416.766 425.367 1.002  1500
## 
## For each parameter, n.eff is a crude measure of effective sample size,
## and Rhat is the potential scale reduction factor (at convergence, Rhat=1).
## 
## DIC info (using the rule, pD = var(deviance)/2)
## pD = 12.7 and DIC = 426.4
## DIC is an estimate of expected predictive error (lower deviance is better).
```

```
autocorr.diag(as.mcmc(mod.pois.zero.adr))
```

```
##              alpha beta_inter beta_moist   beta_temp     deviance   psi_zeros
## Lag 0    1.0000000  1.0000000 1.00000000  1.00000000  1.000000000 1.000000000
## Lag 50   0.9406801  0.9439287 0.96178391  0.90029330 -0.005305089 0.048804233
## Lag 250  0.7432137  0.7484259 0.81660392  0.58658062 -0.001453967 0.036998358
## Lag 500  0.5655857  0.5789891 0.67492710  0.33387486  0.008807748 0.023847308
## Lag 2500 0.1077851  0.1032230 0.05706946 -0.09788742 -0.015919876 0.003910805
##            sigma_a
## Lag 0    1.0000000
## Lag 50   0.5187651
## Lag 250  0.4175428
## Lag 500  0.3570202
## Lag 2500 0.1105911
```

As the Negative binomial model has a lower DIC, all the other models will be discarded.

### Diphascon

For the Diphascon individuals i´ll try two different error families: negative binomial and poisson. As there are no zero´s I won´t test the zero inflated models. The link function is log

Let´s create the jags data:

```
#Data
data_jags=list(Nchamber=12,
               chamber=as.factor(tardigrades_alldata$Chamber),
               individuals=tardigrades_alldata$Diphascon,
               temperature=as.numeric(scale(tardigrades_alldata$csto.avg)),
               moisture=as.numeric(scale(tardigrades_alldata$csm.avg)))
```

And run them:

```
################################################
###### Diphascon with Negative Binomial   ######
################################################

mod_jags_nb <- function(){
  # Priors
  alpha ~ dunif(-1000,1000)# intercept
  beta_temp ~ dunif(-1000,1000)# beta for temperature
  beta_moist ~ dunif(-1000,1000)# beta for soil moisture
  beta_inter ~ dunif(-1000,1000)# beta for interaction
  sigma_a ~ dunif(0, 100) # standard deviation of random effect (variance between sites)
  tau_a <- 1 / (sigma_a * sigma_a) # convert to precision
  r ~ dgamma(0.1 , 0.1) # prior for overdispersion parameter according to: http://doingbayesiandataanalysis.blogspot.com/2012/04/negative-binomial-reparameterization.html
  
  # Random intercept for each chamber (random effect)
  for (j in 1:Nchamber){
    a[j] ~ dnorm(0, tau_a) 
  }
  
  # Likelihood:
  for (i in 1:length(individuals)){
    mu[i] <- exp(alpha + a[chamber[i]] + beta_temp * temperature[i] + beta_moist * moisture[i] + beta_inter * temperature[i] * moisture[i]) #predicted values with log link
    p[i] <- r / ( r + mu[i]) #reparametrization of negbin parameters according to: http://doingbayesiandataanalysis.blogspot.com/2012/04/negative-binomial-reparameterization.html
    individuals[i] ~ dnegbin( p[i] , r )
  }
}

mod_params_nb <- c("alpha", "beta_temp", "beta_moist", "beta_inter", "sigma_a","r")


mod.nb.dip=jags(data=data_jags,parameters.to.save=mod_params_nb, model.file=mod_jags_nb,n.iter=100000)

##################################
###### Diphascon with Poisson ######
##################################

mod_jags_pois <- function(){
  # Priors
  alpha ~ dunif(-1000,1000)# intercept
  beta_temp ~ dunif(-1000,1000)# beta for temperature
  beta_moist ~ dunif(-1000,1000)# beta for soil moisture
  beta_inter ~ dunif(-1000,1000)# beta for interaction
  sigma_a ~ dunif(0, 100) # standard deviation of random effect (variance between sites)
  tau_a <- 1 / (sigma_a * sigma_a) # convert to precision
  
  # Random intercept for each chamber (random effect)
  for (j in 1:Nchamber){
    a[j] ~ dnorm(0, tau_a) 
  }
  
  # Likelihood:
  for (i in 1:length(individuals)){
    mu[i] <- exp(alpha + a[chamber[i]] + beta_temp * temperature[i] + beta_moist * moisture[i] + beta_inter * temperature[i] * moisture[i]) #predicted values with log link
    individuals[i] ~ dpois(mu[i])
  }
}

mod_params_pois <- c("alpha", "beta_temp", "beta_moist", "beta_inter", "sigma_a")


mod.pois.dip=jags(data=data_jags,parameters.to.save=mod_params_pois, model.file=mod_jags_pois,n.iter=100000)
```

Now check if the models converged

Negative binomial

```
mod.nb.dip
```

```
## Inference for Bugs model at "C:/Users/mavecchi/AppData/Local/Temp/RtmpSofaxe/model38545f4277f5.txt", fit using jags,
##  3 chains, each with 1e+05 iterations (first 50000 discarded), n.thin = 50
##  n.sims = 3000 iterations saved
##            mu.vect sd.vect    2.5%     25%     50%     75%   97.5%  Rhat n.eff
## alpha        3.331   0.209   2.943   3.198   3.320   3.465   3.753 1.003   830
## beta_inter   0.468   0.262  -0.026   0.306   0.463   0.629   0.988 1.001  3000
## beta_moist  -0.045   0.216  -0.469  -0.179  -0.042   0.087   0.370 1.001  3000
## beta_temp    0.091   0.222  -0.336  -0.053   0.086   0.224   0.536 1.001  3000
## r            1.786   0.573   0.877   1.382   1.718   2.114   3.088 1.001  3000
## sigma_a      0.306   0.253   0.012   0.120   0.247   0.424   0.921 1.001  2100
## deviance   208.754   4.279 201.956 205.750 208.182 211.173 218.548 1.001  2800
## 
## For each parameter, n.eff is a crude measure of effective sample size,
## and Rhat is the potential scale reduction factor (at convergence, Rhat=1).
## 
## DIC info (using the rule, pD = var(deviance)/2)
## pD = 9.2 and DIC = 217.9
## DIC is an estimate of expected predictive error (lower deviance is better).
```

```
autocorr.diag(as.mcmc(mod.nb.dip))
```

```
##                  alpha  beta_inter   beta_moist     beta_temp     deviance
## Lag 0     1.0000000000  1.00000000  1.000000000  1.0000000000  1.000000000
## Lag 50    0.0048655047 -0.01440897 -0.023180344  0.0000552441 -0.005606192
## Lag 250  -0.0140371398 -0.01265259  0.011006144  0.0240764527 -0.026313599
## Lag 500   0.0003305931 -0.03769129 -0.001784275  0.0066457910 -0.017035082
## Lag 2500  0.0333637094 -0.01308643  0.002563934 -0.0199236204  0.015486719
##                     r      sigma_a
## Lag 0     1.000000000  1.000000000
## Lag 50   -0.013986972  0.223915002
## Lag 250  -0.001269353 -0.019254964
## Lag 500  -0.017799973 -0.030792192
## Lag 2500  0.007847560  0.008205021
```

Poisson

```
mod.pois.dip
```

```
## Inference for Bugs model at "C:/Users/mavecchi/AppData/Local/Temp/RtmpSofaxe/model3854135e2e3c.txt", fit using jags,
##  3 chains, each with 1e+05 iterations (first 50000 discarded), n.thin = 50
##  n.sims = 3000 iterations saved
##            mu.vect sd.vect    2.5%     25%     50%     75%   97.5%  Rhat n.eff
## alpha        3.212   0.168   2.875   3.112   3.214   3.315   3.550 1.003   800
## beta_inter   0.467   0.217   0.045   0.337   0.463   0.595   0.911 1.005   490
## beta_moist  -0.055   0.173  -0.388  -0.164  -0.057   0.048   0.303 1.001  3000
## beta_temp    0.083   0.188  -0.287  -0.026   0.079   0.192   0.451 1.007   290
## sigma_a      0.533   0.183   0.288   0.410   0.496   0.614   0.993 1.001  3000
## deviance   441.004   5.018 433.389 437.336 440.335 443.802 452.545 1.001  2200
## 
## For each parameter, n.eff is a crude measure of effective sample size,
## and Rhat is the potential scale reduction factor (at convergence, Rhat=1).
## 
## DIC info (using the rule, pD = var(deviance)/2)
## pD = 12.6 and DIC = 453.6
## DIC is an estimate of expected predictive error (lower deviance is better).
```

```
autocorr.diag(as.mcmc(mod.pois.dip))
```

```
##                alpha  beta_inter   beta_moist  beta_temp     deviance
## Lag 0     1.00000000 1.000000000  1.000000000 1.00000000  1.000000000
## Lag 50    0.23931554 0.370378074  0.262602072 0.32844682  0.040731285
## Lag 250   0.01004691 0.004634151  0.022856019 0.02891138 -0.035921642
## Lag 500   0.01944565 0.005078747 -0.009023256 0.00786930  0.001491151
## Lag 2500 -0.01151809 0.009182821 -0.025763451 0.02021171  0.014920608
##               sigma_a
## Lag 0     1.000000000
## Lag 50    0.166619923
## Lag 250  -0.006705176
## Lag 500  -0.011386139
## Lag 2500 -0.024425669
```

As the Negative binomial model has a lower DIC, the poisson model will be discarded.

### Mesobiotus

For the Mesobiotus individuals i´ll try two different error families: negative binomial and poisson. As there are no zero´s I won´t test the zero inflated models. The link function is log

Let´s create the jags data:

```
#Data
data_jags=list(Nchamber=12,
               chamber=as.factor(tardigrades_alldata$Chamber),
               individuals=tardigrades_alldata$Mesobiotus,
               temperature=as.numeric(scale(tardigrades_alldata$csto.avg)),
               moisture=as.numeric(scale(tardigrades_alldata$csm.avg)))
```

And run them:

```
################################################
###### Mesobiotus with Negative Binomial     ######
################################################

mod_jags_nb <- function(){
  # Priors
  alpha ~ dunif(-1000,1000)# intercept
  beta_temp ~ dunif(-1000,1000)# beta for temperature
  beta_moist ~ dunif(-1000,1000)# beta for soil moisture
  beta_inter ~ dunif(-1000,1000)# beta for interaction
  sigma_a ~ dunif(0, 100) # standard deviation of random effect (variance between sites)
  tau_a <- 1 / (sigma_a * sigma_a) # convert to precision
  r ~ dgamma(0.1 , 0.1) # prior for overdispersion parameter according to: http://doingbayesiandataanalysis.blogspot.com/2012/04/negative-binomial-reparameterization.html
  
  # Random intercept for each chamber (random effect)
  for (j in 1:Nchamber){
    a[j] ~ dnorm(0, tau_a) 
  }
  
  # Likelihood:
  for (i in 1:length(individuals)){
    mu[i] <- exp(alpha + a[chamber[i]] + beta_temp * temperature[i] + beta_moist * moisture[i] + beta_inter * temperature[i] * moisture[i]) #predicted values with log link
    p[i] <- r / ( r + mu[i]) #reparametrization of negbin parameters according to: http://doingbayesiandataanalysis.blogspot.com/2012/04/negative-binomial-reparameterization.html
    individuals[i] ~ dnegbin( p[i] , r )
  }
}

mod_params_nb <- c("alpha", "beta_temp", "beta_moist", "beta_inter", "sigma_a","r")


mod.nb.mes=jags(data=data_jags,parameters.to.save=mod_params_nb, model.file=mod_jags_nb,n.iter=100000)

##################################
###### Mesobiotus with Poisson ######
##################################

mod_jags_pois <- function(){
  # Priors
  alpha ~ dunif(-1000,1000)# intercept
  beta_temp ~ dunif(-1000,1000)# beta for temperature
  beta_moist ~ dunif(-1000,1000)# beta for soil moisture
  beta_inter ~ dunif(-1000,1000)# beta for interaction
  sigma_a ~ dunif(0, 100) # standard deviation of random effect (variance between sites)
  tau_a <- 1 / (sigma_a * sigma_a) # convert to precision
  
  # Random intercept for each chamber (random effect)
  for (j in 1:Nchamber){
    a[j] ~ dnorm(0, tau_a) 
  }
  
  # Likelihood:
  for (i in 1:length(individuals)){
    mu[i] <- exp(alpha + a[chamber[i]] + beta_temp * temperature[i] + beta_moist * moisture[i] + beta_inter * temperature[i] * moisture[i]) #predicted values with log link
    individuals[i] ~ dpois(mu[i])
  }
}

mod_params_pois <- c("alpha", "beta_temp", "beta_moist", "beta_inter", "sigma_a")


mod.pois.mes=jags(data=data_jags,parameters.to.save=mod_params_pois, model.file=mod_jags_pois,n.iter=100000)
```

Now check if the models converged

Negative binomial

```
mod.nb.mes
```

```
## Inference for Bugs model at "C:/Users/mavecchi/AppData/Local/Temp/RtmpSofaxe/model3854118bbba.txt", fit using jags,
##  3 chains, each with 1e+05 iterations (first 50000 discarded), n.thin = 50
##  n.sims = 3000 iterations saved
##            mu.vect sd.vect    2.5%     25%     50%     75%   97.5%  Rhat n.eff
## alpha        2.205   0.246   1.720   2.050   2.200   2.348   2.708 1.002  1200
## beta_inter   0.531   0.293  -0.028   0.348   0.526   0.721   1.127 1.001  2500
## beta_moist  -0.397   0.265  -0.928  -0.560  -0.393  -0.229   0.128 1.001  3000
## beta_temp    0.176   0.236  -0.286   0.029   0.179   0.327   0.632 1.001  3000
## r            1.599   0.592   0.739   1.178   1.497   1.935   2.974 1.001  3000
## sigma_a      0.352   0.294   0.007   0.129   0.290   0.496   1.065 1.003   850
## deviance   159.262   4.299 152.054 156.391 158.723 161.626 168.796 1.001  3000
## 
## For each parameter, n.eff is a crude measure of effective sample size,
## and Rhat is the potential scale reduction factor (at convergence, Rhat=1).
## 
## DIC info (using the rule, pD = var(deviance)/2)
## pD = 9.2 and DIC = 168.5
## DIC is an estimate of expected predictive error (lower deviance is better).
```

```
autocorr.diag(as.mcmc(mod.nb.mes))
```

```
##                 alpha    beta_inter   beta_moist    beta_temp     deviance
## Lag 0     1.000000000  1.0000000000  1.000000000  1.000000000  1.000000000
## Lag 50    0.007554269 -0.0048824085 -0.006023657 -0.007010716  0.025762378
## Lag 250  -0.019428436 -0.0008176027 -0.012940894  0.021674937 -0.008276654
## Lag 500   0.012901791 -0.0073029580 -0.015567663  0.009660970 -0.001059140
## Lag 2500 -0.015155719 -0.0104518416 -0.012810288 -0.017885176  0.033084269
##                     r      sigma_a
## Lag 0     1.000000000  1.000000000
## Lag 50   -0.002132466  0.236859845
## Lag 250   0.005570756  0.033931923
## Lag 500  -0.018536782 -0.004724981
## Lag 2500 -0.013593803 -0.003586639
```

Poisson

```
mod.pois.mes
```

```
## Inference for Bugs model at "C:/Users/mavecchi/AppData/Local/Temp/RtmpSofaxe/model385485740da.txt", fit using jags,
##  3 chains, each with 1e+05 iterations (first 50000 discarded), n.thin = 50
##  n.sims = 3000 iterations saved
##            mu.vect sd.vect    2.5%     25%     50%     75%   97.5%  Rhat n.eff
## alpha        2.058   0.218   1.610   1.931   2.065   2.194   2.453 1.008  3000
## beta_inter   0.534   0.275  -0.004   0.361   0.530   0.698   1.096 1.001  3000
## beta_moist  -0.395   0.233  -0.859  -0.536  -0.390  -0.255   0.063 1.001  3000
## beta_temp    0.174   0.223  -0.283   0.041   0.175   0.307   0.628 1.001  3000
## sigma_a      0.646   0.246   0.314   0.472   0.602   0.761   1.266 1.001  3000
## deviance   207.120   4.953 199.235 203.502 206.451 210.017 218.550 1.002  1700
## 
## For each parameter, n.eff is a crude measure of effective sample size,
## and Rhat is the potential scale reduction factor (at convergence, Rhat=1).
## 
## DIC info (using the rule, pD = var(deviance)/2)
## pD = 12.3 and DIC = 219.4
## DIC is an estimate of expected predictive error (lower deviance is better).
```

```
autocorr.diag(as.mcmc(mod.pois.mes))
```

```
##                alpha  beta_inter   beta_moist    beta_temp    deviance
## Lag 0     1.00000000  1.00000000  1.000000000  1.000000000 1.000000000
## Lag 50    0.10488949  0.17427705  0.122070788  0.119257586 0.004270223
## Lag 250  -0.01809544 -0.02047176  0.006356333  0.005674605 0.013335107
## Lag 500  -0.03132022 -0.03632846 -0.030432412  0.007291493 0.014127378
## Lag 2500 -0.01108099  0.00186674  0.011537718 -0.008385621 0.000862864
##               sigma_a
## Lag 0     1.000000000
## Lag 50    0.125028652
## Lag 250  -0.004997399
## Lag 500  -0.001520293
## Lag 2500  0.025122919
```

As the Negative binomial model has a lower DIC, the poisson model will be discarded.

##Plotting the estimates

```
inds=data.frame(do.call(rbind,as.mcmc(mod.nb.ind)))[,2:4]
inds=gather(inds,"predictor","value")
inds$response=rep("Number of individuals",nrow(inds))

taxa=data.frame(do.call(rbind,as.mcmc(mod.pois.taxa)))[,2:4]
taxa=gather(taxa,"predictor","value")
taxa$response=rep("Number of taxa",nrow(taxa))

shan=data.frame(do.call(rbind,as.mcmc(mod.sha)))[,2:4]
shan=gather(shan,"predictor","value")
shan$response=rep("Shannon index",nrow(shan))

adr=data.frame(do.call(rbind,as.mcmc(mod.nb.adr)))[,2:4]
adr=gather(adr,"predictor","value")
adr$response=rep("Adropion",nrow(adr))

mes=data.frame(do.call(rbind,as.mcmc(mod.nb.mes)))[,2:4]
mes=gather(mes,"predictor","value")
mes$response=rep("Mesobiotus",nrow(mes))

dip=data.frame(do.call(rbind,as.mcmc(mod.nb.dip)))[,2:4]
dip=gather(dip,"predictor","value")
dip$response=rep("Diphascon",nrow(dip))

toplot=rbind(inds,taxa,shan,adr,mes,dip)

toplot$response=factor(toplot$response,levels=c("Number of individuals","Number of taxa","Shannon index","Adropion","Diphascon","Mesobiotus"))

p=ggplot(toplot)+
  theme_bw() +
  theme(panel.grid.major.x=element_blank(),
        panel.grid.minor.x=element_blank(),
        legend.position = "none",
        strip.background=element_blank(),
        strip.text=element_text(face="bold",size=10),
        plot.title = element_text(hjust = 0.5),
        axis.text.y = element_text())+
  geom_vline(xintercept = 0, col="red",alpha=0.5)+
  stat_density_ridges(aes(x=value,y=predictor,fill=factor(..quantile..)),
                      quantile_lines=T,
                      alpha=0.5,
                      panel_scaling=T,
                      scale=0.9,
                      geom = "density_ridges_gradient", 
                      calc_ecdf = TRUE, 
                      quantiles = c(0.025, 0.975)) +
  scale_fill_manual(name = "Probability", 
                    values = c("#CCCCCCA0", "#73E6FFA0", "#CCCCCCA0"),
                    labels = c("(0, 0.025]", "(0.025, 0.975]", "(0.975, 1]"))+
  scale_y_discrete(labels=c("beta_temp" = "Air temperature", "beta_moist" = "Soil moisture",
                              "beta_inter" = "Air temperature * Soil moisture"))+
  labs(x="Estimated value", y="Predictors",title="Models parameters estimates")+
  facet_wrap(.~response,scales="free_x")
p
```

```
## Picking joint bandwidth of 0.0438
```

```
## Picking joint bandwidth of 0.0221
```

```
## Picking joint bandwidth of 0.0156
```

```
## Picking joint bandwidth of 0.159
```

```
## Picking joint bandwidth of 0.0391
```

```
## Picking joint bandwidth of 0.0452
```

In all the parameters estimates for all the response variables the 0 (red line) falls inside the 95% of the estimated values.  
**No one of the tested predictor is different from zero.**

##Bayesian p-values calculation

```
# Individuals
inds=data.frame(do.call(rbind,as.mcmc(mod.nb.ind)))[,2:4]

pvals_inds = pd_to_p(p_direction(inds)$pd)
names(pvals_inds) = colnames(inds)

es_inds = colMedians(as.matrix((inds/sd(tardigrades_alldata$Individuals))))
names(es_inds) = colnames(inds)


# Taxa
taxa=data.frame(do.call(rbind,as.mcmc(mod.pois.taxa)))[,2:4]

pvals_taxa = pd_to_p(p_direction(taxa)$pd)
names(pvals_taxa) = colnames(taxa)

es_taxa = colMedians(as.matrix((taxa/sd(tardigrades_alldata$Taxa))))
names(es_taxa) = colnames(taxa)

#Shannon
shan=data.frame(do.call(rbind,as.mcmc(mod.sha)))[,2:4]

pvals_shan = pd_to_p(p_direction(shan)$pd)
names(pvals_shan) = colnames(shan)

es_shan = colMedians(as.matrix((shan/sd(tardigrades_alldata$Shannon))))
names(es_shan) = colnames(shan)

# Adropion
adr=data.frame(do.call(rbind,as.mcmc(mod.nb.adr)))[,2:4]

pvals_adr = pd_to_p(p_direction(adr)$pd)
names(pvals_adr) = colnames(adr)

es_adr = colMedians(as.matrix((adr/sd(tardigrades_alldata$Adropion))))
names(es_adr) = colnames(adr)

# Mesobiotus
mes=data.frame(do.call(rbind,as.mcmc(mod.nb.mes)))[,2:4]

pvals_mes = pd_to_p(p_direction(mes)$pd)
names(pvals_mes) = colnames(mes)

es_mes = colMedians(as.matrix((mes/sd(tardigrades_alldata$Mesobiotus))))
names(es_mes) = colnames(mes)

#Diphascon
dip=data.frame(do.call(rbind,as.mcmc(mod.nb.dip)))[,2:4]

pvals_dip = pd_to_p(p_direction(dip)$pd)
names(pvals_dip) = colnames(dip)

es_dip = colMedians(as.matrix((dip/sd(tardigrades_alldata$Diphascon))))
names(es_dip) = colnames(dip)


# Put them together

pvals_dataframe = data.frame(rbind(pvals_inds,
                                   pvals_taxa,
                                   pvals_shan,
                                   pvals_adr,
                                   pvals_mes,
                                   pvals_dip))

pvals_dataframe = pvals_dataframe[,c(3,2,1)]
write.table(pvals_dataframe,file="pvals.txt")


es_dataframe = data.frame(rbind(es_inds,
                                  es_taxa,
                                  es_shan,
                                  es_adr,
                                  es_mes,
                                  es_dip))

es_dataframe = es_dataframe[,c(3,2,1)]
write.table(es_dataframe,file="es.txt")
```

##Appendix 1: worm and density plots of models

```
toplot=data.frame(do.call(rbind,as.mcmc(mod.nb.ind)))
toplot$chain=c(rep("A",1000),rep("B",1000),rep("C",1000))
toplot$timepoint=c(1:1000,1:1000,1:1000)

toplot=gather(toplot,"parameter","value",1:7)

p_worm=ggplot(toplot)+
  geom_line(aes(x=timepoint,y=value,col=chain))+
  facet_grid(parameter~.,scales="free")+
  theme_bw()+
  ggtitle("Individuals - Negative Binomial")
p_dens=ggplot(toplot)+
  geom_density(aes(x=value,col=chain))+
  facet_wrap(parameter~.,scales="free")+
  theme_bw()+ggtitle("Individuals - Negative Binomial")
p_worm
```

```
p_dens
```

```
toplot=data.frame(do.call(rbind,as.mcmc(mod.pois.ind)))
toplot$chain=c(rep("A",1000),rep("B",1000),rep("C",1000))
toplot$timepoint=c(1:1000,1:1000,1:1000)

toplot=gather(toplot,"parameter","value",1:6)

p_worm=ggplot(toplot)+
  geom_line(aes(x=timepoint,y=value,col=chain))+
  facet_grid(parameter~.,scales="free")+
  theme_bw()+
  ggtitle("Individuals - Poisson")
p_dens=ggplot(toplot)+
  geom_density(aes(x=value,col=chain))+
  facet_wrap(parameter~.,scales="free")+
  theme_bw()+ggtitle("Individuals - Poisson")
p_worm
```

```
p_dens
```

```
toplot=data.frame(do.call(rbind,as.mcmc(mod.nb.taxa)))
toplot$chain=c(rep("A",1000),rep("B",1000),rep("C",1000))
toplot$timepoint=c(1:1000,1:1000,1:1000)

toplot=gather(toplot,"parameter","value",1:7)

p_worm=ggplot(toplot)+
  geom_line(aes(x=timepoint,y=value,col=chain))+
  facet_grid(parameter~.,scales="free")+
  theme_bw()+
  ggtitle("Taxa - Negative Binomial")
p_dens=ggplot(toplot)+
  geom_density(aes(x=value,col=chain))+
  facet_wrap(parameter~.,scales="free")+
  theme_bw()+ggtitle("Taxa - Negative Binomial")
p_worm
```

```
p_dens
```

```
toplot=data.frame(do.call(rbind,as.mcmc(mod.pois.taxa)))
toplot$chain=c(rep("A",1000),rep("B",1000),rep("C",1000))
toplot$timepoint=c(1:1000,1:1000,1:1000)

toplot=gather(toplot,"parameter","value",1:6)

p_worm=ggplot(toplot)+
  geom_line(aes(x=timepoint,y=value,col=chain))+
  facet_grid(parameter~.,scales="free")+
  theme_bw()+
  ggtitle("Taxa - Poisson")
p_dens=ggplot(toplot)+
  geom_density(aes(x=value,col=chain))+
  facet_wrap(parameter~.,scales="free")+
  theme_bw()+ggtitle("Taxa - Poisson")
p_worm
```

```
p_dens
```

```
toplot=data.frame(do.call(rbind,as.mcmc(mod.sha)))
toplot$chain=c(rep("A",1000),rep("B",1000),rep("C",1000))
toplot$timepoint=c(1:1000,1:1000,1:1000)

toplot=gather(toplot,"parameter","value",1:7)

p_worm=ggplot(toplot)+
  geom_line(aes(x=timepoint,y=value,col=chain))+
  facet_grid(parameter~.,scales="free")+
  theme_bw()+
  ggtitle("Shannon- Gamma")
p_dens=ggplot(toplot)+
  geom_density(aes(x=value,col=chain))+
  facet_wrap(parameter~.,scales="free")+
  theme_bw()+ggtitle("Shannon- Gamma")
p_worm
```

```
p_dens
```

```
toplot=data.frame(do.call(rbind,as.mcmc(mod.nb.adr)))
toplot$chain=c(rep("A",1000),rep("B",1000),rep("C",1000))
toplot$timepoint=c(1:1000,1:1000,1:1000)

toplot=gather(toplot,"parameter","value",1:7)

p_worm=ggplot(toplot)+
  geom_line(aes(x=timepoint,y=value,col=chain))+
  facet_grid(parameter~.,scales="free")+
  theme_bw()+
  ggtitle("Adropion - Negative Binomial")
p_dens=ggplot(toplot)+
  geom_density(aes(x=value,col=chain))+
  facet_wrap(parameter~.,scales="free")+
  theme_bw()+ggtitle("Adropion - Negative Binomial")
p_worm
```

```
p_dens
```

```
toplot=data.frame(do.call(rbind,as.mcmc(mod.nb.zero.adr)))
toplot$chain=c(rep("A",1000),rep("B",1000),rep("C",1000))
toplot$timepoint=c(1:1000,1:1000,1:1000)

toplot=gather(toplot,"parameter","value",1:8)

p_worm=ggplot(toplot)+
  geom_line(aes(x=timepoint,y=value,col=chain))+
  facet_grid(parameter~.,scales="free")+
  theme_bw()+
  ggtitle("Adropion - zero inflated Negative Binomial")
p_dens=ggplot(toplot)+
  geom_density(aes(x=value,col=chain))+
  facet_wrap(parameter~.,scales="free")+
  theme_bw()+ggtitle("Adropion - zero inflated Negative Binomial")
p_worm
```

```
p_dens
```

```
toplot=data.frame(do.call(rbind,as.mcmc(mod.pois.adr)))
toplot$chain=c(rep("A",1000),rep("B",1000),rep("C",1000))
toplot$timepoint=c(1:1000,1:1000,1:1000)

toplot=gather(toplot,"parameter","value",1:6)

p_worm=ggplot(toplot)+
  geom_line(aes(x=timepoint,y=value,col=chain))+
  facet_grid(parameter~.,scales="free")+
  theme_bw()+
  ggtitle("Adropion - Poisson")
p_dens=ggplot(toplot)+
  geom_density(aes(x=value,col=chain))+
  facet_wrap(parameter~.,scales="free")+
  theme_bw()+ggtitle("Adropion - Poisson")
p_worm
```

```
p_dens
```

```
toplot=data.frame(do.call(rbind,as.mcmc(mod.pois.zero.adr)))
toplot$chain=c(rep("A",1000),rep("B",1000),rep("C",1000))
toplot$timepoint=c(1:1000,1:1000,1:1000)

toplot=gather(toplot,"parameter","value",1:7)

p_worm=ggplot(toplot)+
  geom_line(aes(x=timepoint,y=value,col=chain))+
  facet_grid(parameter~.,scales="free")+
  theme_bw()+
  ggtitle("Adropion - zero inflated Poisson")
p_dens=ggplot(toplot)+
  geom_density(aes(x=value,col=chain))+
  facet_wrap(parameter~.,scales="free")+
  theme_bw()+ggtitle("Adropion - zero inflated Poisson")
p_worm
```

```
p_dens
```

```
toplot=data.frame(do.call(rbind,as.mcmc(mod.nb.mes)))
toplot$chain=c(rep("A",1000),rep("B",1000),rep("C",1000))
toplot$timepoint=c(1:1000,1:1000,1:1000)

toplot=gather(toplot,"parameter","value",1:7)

p_worm=ggplot(toplot)+
  geom_line(aes(x=timepoint,y=value,col=chain))+
  facet_grid(parameter~.,scales="free")+
  theme_bw()+
  ggtitle("Mesobiotus - Negative Binomial")
p_dens=ggplot(toplot)+
  geom_density(aes(x=value,col=chain))+
  facet_wrap(parameter~.,scales="free")+
  theme_bw()+ggtitle("Mesobiotus - Negative Binomial")
p_worm
```

```
p_dens
```

```
toplot=data.frame(do.call(rbind,as.mcmc(mod.pois.mes)))
toplot$chain=c(rep("A",1000),rep("B",1000),rep("C",1000))
toplot$timepoint=c(1:1000,1:1000,1:1000)

toplot=gather(toplot,"parameter","value",1:6)

p_worm=ggplot(toplot)+
  geom_line(aes(x=timepoint,y=value,col=chain))+
  facet_grid(parameter~.,scales="free")+
  theme_bw()+
  ggtitle("Mesobiotus - Poisson")
p_dens=ggplot(toplot)+
  geom_density(aes(x=value,col=chain))+
  facet_wrap(parameter~.,scales="free")+
  theme_bw()+ggtitle("Mesobiotus - Poisson")
p_worm
```

```
p_dens
```

```
toplot=data.frame(do.call(rbind,as.mcmc(mod.nb.dip)))
toplot$chain=c(rep("A",1000),rep("B",1000),rep("C",1000))
toplot$timepoint=c(1:1000,1:1000,1:1000)

toplot=gather(toplot,"parameter","value",1:7)

p_worm=ggplot(toplot)+
  geom_line(aes(x=timepoint,y=value,col=chain))+
  facet_grid(parameter~.,scales="free")+
  theme_bw()+
  ggtitle("Diphascon - Negative Binomial")
p_dens=ggplot(toplot)+
  geom_density(aes(x=value,col=chain))+
  facet_wrap(parameter~.,scales="free")+
  theme_bw()+ggtitle("Diphascon - Negative Binomial")
p_worm
```

```
p_dens
```

```
toplot=data.frame(do.call(rbind,as.mcmc(mod.pois.dip)))
toplot$chain=c(rep("A",1000),rep("B",1000),rep("C",1000))
toplot$timepoint=c(1:1000,1:1000,1:1000)

toplot=gather(toplot,"parameter","value",1:6)

p_worm=ggplot(toplot)+
  geom_line(aes(x=timepoint,y=value,col=chain))+
  facet_grid(parameter~.,scales="free")+
  theme_bw()+
  ggtitle("Diphascon - Poisson")
p_dens=ggplot(toplot)+
  geom_density(aes(x=value,col=chain))+
  facet_wrap(parameter~.,scales="free")+
  theme_bw()+ggtitle("Diphascon - Poisson")
p_worm
```

```
p_dens
```

NB: in the Negative Binomial on number of Taxa the r parameter is really large and almost take the shape of the prior distribution. I tried to increase the prior from dunif(0,100) to dunif(0,1000), however the posterior distribution span all of it anyway. This is not really a problem as with high r values, the Negative Binomial distribution approximates a Poisson distribution that was found to have a lower DIC.

##Appendix 2: ptable with number of individuals by taxa found in each chamber and temperature and soil moisture data
